# A single-stranded DNA virus replicates in the mitochondria of a marine oomycete

**DOI:** 10.1093/ve/veag047

**Published:** 2026-07-31

**Authors:** Kohei Sakuta, Mart Krupovic, Ondřej Hejna, Cristiana Maia, Marília Horta Jung, Ken Komatsu, Hiromitsu Moriyama, Thomas Jung, Leticia Botella

**Affiliations:** Laboratory of Molecular and Cellular Biology, Graduate School of Agriculture, Tokyo University of Agriculture and Technology, 3-5-8 Saiwaicho, Fuchu, Tokyo, 183-8509, Japan; Department of Life Sciences, Graduate School of Arts and Sciences, The University of Tokyo, 3-8-1, Komaba, Meguro-ku, Tokyo 153-8902, Japan; Institut Pasteur, Université Paris Cité, Cell Biology and Virology of Archaea Unit, 25 rue du Dr. Roux, 75015, Paris, France; Department of Genetics and Agrobiotechnology, Faculty of Agriculture and Technology, University of South Bohemia in České Budějovice, Na Sádkách 1780, 370 05 České Budějovice, Czech Republic; Centre of Marine Sciences (CCMAR), University of Algarve, Gambelas Campus 8005-139, Faro 8005-139, Portugal; Phytophthora Research Centre, Department of Forest Protection and Wildlife Management, Faculty of Forestry and Wood Technology, Mendel University in Brno, Zemědělská 3, 613 00 Brno, Czech Republic; Laboratory of Molecular and Cellular Biology, Graduate School of Agriculture, Tokyo University of Agriculture and Technology, 3-5-8 Saiwaicho, Fuchu, Tokyo, 183-8509, Japan; Laboratory of Molecular and Cellular Biology, Graduate School of Agriculture, Tokyo University of Agriculture and Technology, 3-5-8 Saiwaicho, Fuchu, Tokyo, 183-8509, Japan; Phytophthora Research Centre, Department of Forest Protection and Wildlife Management, Faculty of Forestry and Wood Technology, Mendel University in Brno, Zemědělská 3, 613 00 Brno, Czech Republic; Phytophthora Research Centre, Department of Forest Protection and Wildlife Management, Faculty of Forestry and Wood Technology, Mendel University in Brno, Zemědělská 3, 613 00 Brno, Czech Republic; Department of Forest Protection and Wildlife Management, Faculty of Forestry and Wood Technology, Mendel University in Brno, Zemědělská 3, 613 00 Brno, Czech Republic

**Keywords:** *Cressdnaviricota*, HUH superfamily rolling-circle replication endonuclease, Oomycota, mitochondrial integration, viral endogenization, chondriosomes, respiration

## Abstract

A virus with a circular single-stranded DNA genome, HthCRESSV1, was discovered in an oomycete, *Halophytophthora thermoambigua*, isolated from brackish waters off the Algarve coast in southern Portugal. Phylogenetic analyses place this virus outside of all currently classified families within the phylum *Cressdnaviricota*, suggesting it represents a distinct lineage. Cellular fractionation, mitochondrial marker co-enrichment, and Southern blot analyses indicate that HthCRESSV1 is associated with mitochondria and replicates episomally. The virus is stably transmitted through zoospores and is associated with a reduced host mycelial growth. Homologous sequences corresponding to both the replication-associated protein and a membrane-associated hypothetical protein were identified in the mitochondrial genomes of several oomycete species, suggesting recurrent endogenization of HthCRESSV1-like viruses in oomycete mitochondria. Together, these findings expand the known diversity of oomycete-associated DNA viruses, identify mitochondria as an unexpected niche for DNA virus replication, and highlight marine environments as underexplored reservoirs of DNA viruses in stramenopiles.

## 1. Introduction

Metagenomic analyses have revealed that viruses with single-stranded DNA (ssDNA) genomes are ubiquitous, remarkably diverse, and infect cellular organisms across the tree of life (Rosario et al. 2012, Krupovic 2013, Zhao et al. 2019). One of the most diverse groups of ssDNA viruses is represented by members of the phylum *Cressdnaviricota* (Krupovic et al. 2020a), formerly known as the circular, Rep-encoding single-stranded DNA (CRESS DNA) viruses (Rosario et al. 2012). These viruses share the characteristic two-domain rolling circle replication (RCR) initiation protein (Rep) comprising the N-terminal HUH superfamily endonuclease domain and the C-terminal superfamily 3 helicase domain (Kazlauskas et al. 2019). All cressdnaviricots characterized thus far build nonenveloped, icosahedral capsids and encode CPs with the jelly-roll fold (antiparallel 8-strand β-barrel) (Krupovic et al. 2020a, Kulshrestha et al. 2020). Whereas the Rep is universally encoded across *Cressdnaviricota*, the capsid protein (CP) genes of these viruses display considerable sequence divergence and in some lineages have been demonstrated to undergo gene replacement with nonorthologous but structurally related CPs from other viruses (Diemer and Stedman 2012, Roux et al. 2013, Kazlauskas et al. 2017, Munke et al. 2022). Thus, the phylogenetic analysis of the Rep proteins is used as a framework for the classification of cressdnaviricots into families, orders, and classes.

Currently, *Cressdnaviricota* comprises two classes (*Arfiviricetes* and *Repensiviricetes*), 13 orders, and 24 families (Simmonds et al. 2025). All cressdnaviricots for which the hosts have been experimentally determined infect eukaryotes, including plants (e.g. *Nanoviridae*, *Geminiviridae*, *Amesuviridae*, *Metaxyviridae*), fungi (*Genomoviridae* [also shown to replicate in insects]), animals (e.g. *Circoviridae*, *Redondoviridae*), algae (*Bacilladnaviridae*), and protists (e.g. *Naryaviridae*, *Nenyaviridae*, *Vilyaviridae, Oomyviridae*) (Krupovic et al. 2020a,b, Varsani and Krupovic 2024, Varsani et al. 2024). However, hosts of cressdnaviricot viruses representing a considerable fraction of families are currently unknown. Furthermore, many ssDNA viruses, especially those discovered through metagenomics, remain unclassified. Thus, the extent of cressdnaviricot diversity and their distribution in eukaryotes remain to be fully appreciated.

Several ssDNA viruses have been described in economically important plant pathogenic filamentous fungi, including *Sclerotinia sclerotiorum* (infected by Sclerotinia sclerotiorum hypovirulence-associated DNA virus 1, SsHADV1), *Fusarium graminearum* (Fusarium graminearum gemytripvirus 1, FgGMTV1), *Diaporthe sojae* (Diaporthe sojae circular DNA virus 1, DsCDV1), and *Botrytis cinerea* (Botrytis cinerea ssDNA virus 1, BcssDV1) (Yu et al. 2010, Li et al. 2020, Hao et al. 2021, Ruiz-Padilla et al. 2023, Wang et al. 2024). No ssDNA viruses infecting oomycetes have been isolated but metagenomic studies have uncovered the existence of multiple endogenous viral elements (EVEs), encompassing the Rep and CP genes typical of ssDNA viruses, in the genomes of oomycetes (Sabanadzovic et al. 2025). In particular, EVEs were found in chromosome 5 of *Phytophthora plurivora*, *P. parasitica* (synonym *P. nicotianae*), *P. fragariae*, *Aphanomyces astaci,* and *Pythium insidiosum*. Furthermore, transcripts corresponding to ssDNA virus genes were detected in the transcriptomes of *Plasmopara halstedii* and *A. invadans*, suggesting the presence of transcriptionally active viral elements and raising the possibility of ongoing or recent viral replication rather than solely degenerated genomic remnants (Sabanadzovic et al. 2025). Environmental metatranscriptomic analyses have further identified diverse cressdnaviricot sequences in various habitats known to harbour fungi and oomycetes, including soil, riverbed sediments, plant samples, and animal faeces (Eaglesham and Hewson 2013, Reavy et al. 2015, Rosario et al. 2018, Fehér et al. 2021, Varsani and Krupovic 2021, Zhao et al. 2021). Therefore, ssDNA viruses of fungi and oomycetes may play important, yet underappreciated roles in ecosystem structuring by controlling the behaviour and dynamics of their hosts (Kondo et al. 2022). Indeed, several SsHADV1-like ssDNA viruses (family *Genomoviridae*) have been shown to induce hypovirulence of their phytopathogenic fungal hosts (Yu et al. 2010, Li et al. 2020).

In marine ecosystems, viral infections are observed in all organisms from bacteria to whales, and the presence of these viruses influences marine biological community composition and serves as a potential driving force in biogeochemical cycles (Suttle 2005). Although dsDNA phages infecting bacteria are the most abundant viruses in marine ecosystems, metagenomic and metatranscriptomic studies have uncovered a substantial contribution to the global marine virome of ssDNA and RNA viruses that infect protists, including unicellular algae, fungi, and fungus-like oomycetes (Coy et al. 2018). RNA viruses have been identified from various host fungi infecting *Posidonia oceanica* (seagrass) and *Holothuria poli*, and oomycetes, including *Phytophthora condilina* and *Halophytophthora frigida* (Nerva et al. 2016, 2019, Botella et al. 2020, Botella and Jung 2021). However, no DNA viruses infecting marine fungi or oomycetes have been identified to date.

The genus *Halophytophthora* constitutes a sister genus of the well-known plant pathogenic oomycete genus *Phytophthora*, comprising species with similar morphology and life cycles, almost exclusively inhabiting brackish and saltwater environments (Sullivan et al. 2018). While some species, such as *H. lateralis* (previously *Halophytophthora* sp. Zostera), have been demonstrated to be pathogenic to eelgrass (*Zostera marina*) (Govers et al. 2016), the genus *Halophytophthora* has been primarily considered as decomposers in mangrove ecosystems (Newell and Fell 1992, 1997). The only report of viral infection in this genus is the co-infection of eight bunya-like viruses in *H. frigida* collected from estuaries in southern Portugal, and the effects of viral infection on the host remain unclear (Botella et al. 2020).

To expand the limited knowledge on viruses in *Halophytophthora*, we focused our viral discovery and characterization efforts on *H. thermoambigua*, a species inhabiting marine and brackish-water environments (Maia et al. 2022). *Halophytophthora thermoambigua* emerged as a compelling candidate for virus screening due to two notable biological features: its self-sterile breeding system, with no observed oogonia, oospores, or antheridia, and its unusual phenotypic variability in colony morphology and optimal growth temperature among isolates (20°C, 25°C, or 27.5°C). Here, we report the discovery of the first DNA virus identified in an oomycete. We demonstrate its mitochondrial localization, confirm its extrachromosomal replicative form, and provide evidence of stable vertical transmission. The virus is associated with reduced host growth and altered temperature-dependent performance.

## 2. Materials and methods

### 2.1. Strains and culture conditions

All *Halophytophthora* isolates/strains investigated in this study were collected in December 2015 from seven different marine and brackish water sites along the Algarve coast of southern Portugal using an *in situ* baiting method (Jung et al. 2017) in salt marshes, tidal pools, channels, lagoons, and estuaries that remained submerged even at low tide (Maia et al. 2022). Supplementary Table S1 provides detailed information and species identification for all *Halophytophthora* isolates included in the experiments. All strains were cultured on salty V8-agar medium (sV8A; 16 g agar, 3 g CaCO3, 100 ml Campbell’s V8 juice, 450 ml distilled water, 450 ml seawater (Maia et al. 2022); or liquid V8-medium containing seawater adjusted to 50% concentration (sV8).

### 2.2. RNA extraction

Total RNA was purified from ~100 mg of 7–10-days old mycelium using RNAzol® RT Column Kit (Chomczynski et al. 2010) and treated with TURBO DNA-free™ Kit (Ambion). RNA was quantified in Qubit® 2.0 Fluorometer (Invitrogen), and quality was tested by Tape Station 4200 (Agilent). One RNA pool was prepared with eight isolates derived of the original ex-type species of *Halophytophthora* described in Maia et al. (2022).

### 2.3. RNA library preparation and total stranded RNA sequencing

Around 1 μg of total RNA, eluted in RNase-free water, was sent to IABio in Olomouc, Czech Republic, for RNA library construction and deep sequencing. Ribosomal RNA (rRNA) was depleted using the NEBNext rRNA Depletion Kit (Human/Mouse/Rat) and prepared with the NEBNext Ultra II Directional RNA Library Prep Kit for Illumina, along with NEBNext Multiplex Oligos for Illumina (Unique Dual Index Primer Pairs). Library quality control was evaluated with the Agilent Bioanalyzer 2100 High Sensitivity DNA Kit. The KAPA Library Quantification Kit for the Illumina platform facilitated absolute quantitative PCR (qPCR)-based quantification of the Illumina libraries, flanked by the P5 and P7 flow cell oligo sequences. The libraries underwent paired-end (PE) (2 × 150 nt) sequencing on a NovaSeq6000 (DS-150) from Illumina, San Diego, CA, USA, using the NovaSeq S4 v1.5 reagent kit. An ‘in-lane’ PhiX control spike was included in each lane of the flow cell.

### 2.4. Virus identification bioinformatics pipeline

#### 2.4.1. Preprocessing of RNA-seq data

Raw data were processed by BaseSpace cloud interface (Illumina) in default settings. The processing was carried out on the local server of the University of South Bohemia in České Budějovice, Czech Republic. The basecalling, adapter clipping, and quality filtering were carried out using Bcl2fastq v2.20.0.422 Conversion Software (Illumina). For the initial quality assessment, we used FastQC v0.11.9 (https://www.bioinformatics.babraham.ac.uk/projects/fastqc/). The adapter sequences, provided by the sequencing firm, were verified using BBTools v37.87 (https://jgi.doe.gov/data-and-tools/software-tools/bbtools/) (Bushnell et al. 2017). Following processing with Cutadapt v4.7 (https://cutadapt.readthedocs.io/en/stable/) (Martin 2011) involved trimming of N bases, adapter sequences, and low-quality ends (<30). Reads falling short of 50 bp were discarded from subsequent analyses. Post-trimming quality was again re-assessed with FastQC. The remaining rRNA reads were removed using SortMeRNA v4.3.6 (https://github.com/sortmerna/sortmerna/) (Kopylova et al. 2012). SILVA SSU and LSU databases filtered to include only Oomycota rRNA sequences were used as references (https://www.arb-silva.de/).

#### 2.4.2. Alignment/read mapping to host genome

For mapping reads to the host genome, the STAR v2.7.9a (Dobin 2014) program was employed with default settings. Two genomes, *H. batemanensis* (GCA_023338225.1) and

*H. polymorphica* (GCA_023338205.1), were used as references. After alignment to the reference sequence, all mapped reads were discarded and only unmapped reads were utilized further.

#### 2.4.3. *De novo* assembly of unmapped reads for detection of novel viruses

The unmapped reads were used for *de novo* assembly. To assemble these reads into contigs, the SPAdes program v3.15.5 (https://github.com/ablab/spades/) (Bankevich et al. 2012) was used with default settings for metagenomics. For further analysis, assembled contigs shorter than 500 bp were discarded.

#### 2.4.4. Search for similarity in virus databases

Final contigs were cross-referenced against several databases using the Basic Local Alignment Search Tool (BLAST) v2.10.0 (https://blast.ncbi.nlm.nih.gov/doc/blast-help/downloadblastdata.html/ (Camacho et al. 2009). As a reference, we used viral reference sequences from NCBI. BLASTn was applied for the nucleotide database accessible at https://www.ncbi.nlm.nih.gov/labs/virus/vssi/#/virus?SeqType_s=Nucleotide. Similarly, BLASTx was employed for the protein database available at https://www.ncbi.nlm.nih.gov/labs/virus/vssi/#/virus?SeqType_s=Protein and also to search the UniProt database for a specified virus taxon at https://www.uniprot.org/uniprot/?query=taxonomy:10239. Contigs with an e-value higher than 1e-3 were discarded. Subsequently, the remaining contigs were processed in a similar manner using the entire range of databases. For BLASTn, the NCBI nucleotide (nt) database from NCBI, available at https://ftp.ncbi.nlm.nih.gov/blast/db/nt*, was used. For BLASTx, both the NCBI nr database, accessible at https://ftp.ncbi.nlm.nih.gov/blast/db/nr*, and the UniProt Knowledgebase (UniProtKB) available at https://www.uniprot.org/uniprotkb/, were employed. The BLAST search was restricted to the best hit per contig. The final list of candidate viral contigs was compiled by scanning the BLAST results for the search string ‘vir’.

To identify homologous replicases and hypothetical proteins that might not be annotated in Blastx, a BLASTn search against the NCBI nt database using the full-length HthCRESSV1 genomic sequence as a query. Hits with high nucleotide similarity (e-value <1e−10) were retrieved and manually inspected. Top matches corresponded to mitochondrial genome assemblies from diverse oomycete species. To confirm the genomic context and sequence identity, the HthCRESSV1-Rep and HP sequences were mapped to these mitochondrial genome assemblies using Geneious Prime (v2023.2.2), employing medium sensitivity and default parameters.

#### 2.4.5. Coverage depth

The number of unique sequencing reads that align to each reference *de novo* assembled viral contig was calculated with the following formula: (Total reads mapped to the final identified virus * average read length)/virus genome or contig length) (Sims et al. 2014). Coverage plots were visualized with Integrated Genome Viewer (IGV) tool and Geneious Prime® 2025.0.2. (Figure S1).

### 2.5. DNA extraction and PCR reactions

Purified DNA used for Southern hybridization was extracted using the modified cetyltrimethylammonium bromide (CTAB)-based method by Fukuhara et al. (1993). Specifically, ~0.1 g of dried mycelia harvested after 7 days of culture in sV8 liquid medium was used as starting material, and the extraction was performed on a small scale. DNA extraction from mycelia after single lesion isolation derived from zoospores was performed as described by Maia et al. (2022). DNA was stored at −80°C for long-term preservation. PCR reactions for complete sequence determination and detection of HtCRESSV1 were performed using the obtained DNA as template with primers listed in Table S2 and GoTaq (Promega, Wisconsin, USA).

### 2.6. Phylogenetic analyses

Maximum likelihood (ML) phylogenetic trees were constructed using RAxML-HPC v.8 on XSEDE *via* the CIPRES Science Gateway (Stamatakis 2006). The analysis employed a rapid bootstrapping algorithm with the recommended parameters, and the GAMMA model was used to avoid extensive optimization of the best-scoring ML tree at the end of the run. The Jones–Taylor–Thornton (JTT) model was applied as the amino acid (aa) substitution model for protein sequences.

The resulting trees were visualized using iTOL v6 (Letunic and Bork 2021) (https://itol.embl.de).

### 2.7. Stem loop prediction

DNA secondary structure prediction and free energy calculations were carried out using two web-based computational tools. The mFold web server (hosted by UNAFold) was used to predict and visualize DNA secondary structures and determine associated thermodynamic parameters, including minimum free energy, under defined temperature and ionic conditions (https://www.unafold.org/mfold/applications/Structure-display-and-free-energy-determination.php). Additionally, the ViennaRNA Web Services platform (http://rna.tbi.univie.ac.at/) was also employed for further structure prediction and analysis. Although primarily designed for RNA, the ViennaRNA tools were adapted for DNA by specifying the appropriate nucleic acid type and parameters where applicable.

### 2.8. Analysis of the ORF2 protein sequence and structure

Secondary structure and putative transmembrane domains were predicted using the PSIPRED and MEMSAT-SVM algorithms, respectively, available through the PSIPRED Workbench (Buchan et al. 2024). Coiled-coil regions were predicted using PCOILS available through the MPI Bioinformatics Toolkit (Zimmermann et al. 2018). Structural modelling of ORF2 protein sequence was carried out using AlphaFold3 (Abramson et al. 2024). The models were built for different oligomeric states from monomer to homo-decamer. The quality of the models was evaluated by comparing their predicted template modelling (pTM) and the interface predicted template modelling (ipTM) scores. The resulting models were visualized using ChimeraX v1.11.1 (Meng et al. 2023).

### 2.9. Virus-like particle purification and transmission electron microscopy observation

Approximately 40 g of BD651 mycelium cultured in liquid s. V8 medium was collected using Miracloth, washed with distilled water, and incubated in Buffer A (0.1 M phosphate buffer, 0.2 M KCl) for 10 min. The mycelium was collected, homogenized in liquid nitrogen, and disrupted using a French press. Triton X-100 (1% final concentration) was added to the cell lysate, followed by stirring at 4°C for 30 min. The lysate was centrifuged at 1000 *g* for 10 min to remove cellular debris. The supernatant was collected, mixed with an equal volume of chloroform, gently agitated for 30 min, and centrifuged at 8000 *g* for 15 min. The aqueous phase was collected and centrifuged at 20 000 *g* for 30 min to pellet heavy membrane fractions. The resulting supernatant was ultracentrifuged at 100 000 *g* for 1 h using a Hitachi CP80WX P45AT rotor to pellet virus-like particles (VLPs; P100 000). The pellet was resuspended in Buffer A and subjected to continuous sucrose density gradient centrifugation (10%–40% w/v) at 80 000 *g* for 3 h at 4°C using a Hitachi CP80WX P28S swing rotor. Following ultracentrifugation, the gradient was fractionated from the top in 2 ml aliquots using a Hitachi DGF-U gradient fractionator, yielding 15 fractions. Total nucleic acids were extracted from each fraction, and HthCRESSV1-enriched fractions (fractions 4 and 5) were identified by HthCRESSV1-specific PCR. These fractions were pooled and concentrated by ultracentrifugation at 100 000 *g* for 1 h using a Hitachi CP80WX P45AT rotor. The pellet was resuspended in phosphate buffer, negatively stained with 2% uranyl acetate, and visualized by transmission electron microscopy (TEM) using a JEM-1400 Plus microscope (JEOL, Tokyo, Japan).

### 2.10. Southern blot analysis

Approximately 5 μg of purified DNA extracted from strain BD651 were subjected to electrophoresis on a 0.8% agarose gel, followed by depurination treatment by immersing the gel in 0.25 M HCl and shaking for 5 min. After washing the gel with distilled water, DNA was denatured by shaking in denaturing solution (0.5 M NaOH, 1.5 M NaCl) for 15 min. This operation was performed twice. The denatured gel was then neutralized by shaking in neutralizing solution (0.5 M Tris–HCl, pH 7.5, 1.5 M NaCl) for 15 min, repeated twice, washed with distilled water, and equilibrated by incubation in 20× SSC for 10 min. Subsequently, the DNA was transferred to a positively charged nylon membrane (Roche, Basel, Switzerland) by capillary blotting. To detect the HtCRESSV1 genome, a DIG-labelled DNA probe was prepared using the HtCRESSV1-HP probe (nt 114–837) according to the protocol provided with the PCR DIG Labeling Mix (Roche, Basel, Switzerland). Hybridization with the DNA probe was performed at 50°C for 16 h. Subsequent operations until detection were performed according to the method of Sakuta et al. (2023).

### 2.11. Mitochondrial purification and nucleic acid extraction

Approximately 20 g of mycelia were homogenized in 200 ml of isotonic solution (0.25 M sucrose, 100 mM Tris–HCl, pH 7.4, 10 mM EGTA). The homogenate was transferred to a 50 ml centrifuge tube and centrifuged at 1000 × *g* for 10 min to precipitate cell debris and nuclei (P1000). The supernatant was transferred to a Hizacks tube and centrifuged at 12 000 × *g* for 15 min to obtain a crude mitochondrial fraction enriched in mitochondria (P12 000). This supernatant was used as a membrane fraction and centrifuged at 100 000 × *g* for 90 min using a P45AT rotor in a Hitachi CP80WX (Hitachi Koki, Japan), with the resulting pellet designated as the membrane fraction (P100 000) and the supernatant as the cytosolic fraction (S100 000). P12 000 was purified using two rounds of sucrose density gradient centrifugation to obtain a more intact mitochondrial fraction. First, it was layered on a discontinuous sucrose solution prepared at concentrations of 15%–23%–32%–60% and centrifuged at 82 200 × *g* for 3 h using a P28S swing rotor in a Hitachi CP80WX (Hitachi Koki, Japan). After centrifugation, the purified mitochondrial concentrated fraction observed at the 32%–60% interface was carefully collected, diluted with isotonic solution, centrifuged at 10 000 × *g* for 20 min, and the resulting pellet was resuspended in isotonic solution to obtain a semi-purified mitochondrial fraction. The second purification was performed by layering the semi-purified mitochondrial fraction on a sucrose solution prepared at 32%–60% concentration and ultracentrifuging under the same conditions as the first round. After centrifugation, 2 ml fractions were carefully collected from the top to obtain 15 fractions (F1–F15). Total DNA was extracted from the 15 fractions containing purified mitochondrial fractions using a modified method of Okada et al. (2015). Specifically, samples were mixed with 0.5 ml of 2× Saline-Tris-EDTA (STE) buffer (200 mM NaCl, 20 mM Tris–HCl pH 8.0, 2 mM EDTA pH 8.0) containing 1% sodium dodecyl sulfate (SDS) and 0.5 ml of 1:1 phenol/chloroform. The mixture was vortexed and centrifuged at 15 000 *g* for 5 min. The resulting supernatant was used as a total nucleic acid sample, and after ethanol precipitation, it was suspended in TE (1M Tris-HCl, 0.5M EDTA, pH 8.0) buffer containing RNase A and incubated at 37°C for 30 min to obtain a total DNA sample.

### 2.12. COX activity measurement

To measure the activity of cytochrome c oxidase, a mitochondrial marker enzyme, the Cytochrome c Oxidase Assay Kit (Sigma) was used according to the manufacturer’s instructions. Enzyme activity was measured by monitoring the absorbance at 550 nm for 1 min, with the decrease in absorbance defined as ΔA and substituted into the following equation to measure enzyme activity: Units/ml·g = ΔA/min × 1.1/(0.1 × 21.84 × X) (X: weight of source mycelia in grams).

### 2.13. Zoospore induction and single lesion isolation, mycelial comparison, and growth tests

Zoospore induction of *H. thermoambigua* was performed using modified protocols from Jesus et al. (2016) and Jung et al. (1999). Single lesion formation was achieved by floating young cork oak (*Quercus suber*) leaves on the water surface in nonsterile 50% seawater after zoospore induction and incubating at 20°C under natural light for 24 h. The single lesions were excised with a razor blade and placed on PARPNH agar medium (V8 juice agar (V8A) supplemented with 10 μg/ml pimaricin, 200 μg/ml ampicillin, 10 μg/ml rifampicin, 25 μg/ml pentachloronitrobenzene (PCNB), 50 μg/ml nystatin, and 50 μg/ml hymexazol) to obtain single zoospore-derived isolates. The relationship between temperature and growth was examined by preculturing target strains on 90 mm V8A medium for 1 week to standardize the growth stage. Subsequently, three plates per isolate were incubated at 4°C, 8°C, 15°C, 20°C, 25°C, 28°C, 30°C, and 33°C in the dark. The test was conducted for 5 days or until the colonies nearly reached the edge of the Petri dish, and the average growth rate (cm/day) was calculated.

### 2.14. Attempted HthCRESSV1 elimination

A total of 20 single-zoospore strains derived from the isolate BD651 were prepared, following the procedure described by Uchida et al. (2021) and Sakuta et al. (2025). The regeneration medium was supplemented with ribavirin (300 μg ml^−1^ final concentration) and cycloheximide (5 μg ml^−1^ final concentration), and 20 isolates were subsequently recovered from the regenerated mycelia. Total nucleic acids were then extracted from each culture grown on sV8 medium using the phenol–chloroform–isoamyl alcohol (PCI) method. The presence of the virus was finally verified by PCR employing a primer set specific to HtCRESSV1 (Table S2).

### 2.15. Statistical analysis

Colony diameter measurement data were subjected to analysis of variance. Dunnett’s test following one-way analysis of variance was applied for comparison of means between virus-infected and noninfected strain groups. Statistical significance was set at *P* < .05 (*P < .05, **P < .01).

## 3. Results

### 3.1. Identification of Halophytophthora thermoambigua CRESS virus 1

Transcriptomic screening of eight pooled strains of *Halophytophthora* spp. (Table S1) yielded 729 sequencing reads that could be assembled into a 979 nt-long contig (NODE_2192_length_979; Fig. S1) that displayed similarity to unclassified ssDNA viruses, including Tick-associated circular DNA virus (UTM74968.1; 27.6% identity, 76.3% query coverage, *E* = 2.69E-11) and *Grus japonensis* CRESS-DNA-virus sp. (QTE03368.1; 26.9% identity, 71.2% coverage, *E* = 1.17E-09). The hits to viruses encompassed a region encoding the Gemini_AL1 domain (Pfam: PF00799; *E*-value: 2.26e-07) corresponding to the catalytic domain of the geminiviral replication initiation protein (Rep), suggesting that the assembled contig corresponds to a previously unknown member of the phylum *Cressdnaviricota*. Notably, a BLASTx search queried with the contig sequence identified as the top hit a hypothetical protein (N0F65_000360) encoded by the mitochondrial genome of the oomycete *Lagenidium giganteum* (accession DAZ93511.1), with 67.81% identity and 45% query coverage (*E*-value = 4e-63). The second and third best hits were also to two mitochondrially encoded hypothetical proteins from *P. sojae* (YP_001165403 and YP_001165402), pointing to the association of the identified virus with different oomycete species.

**Figure 1:**
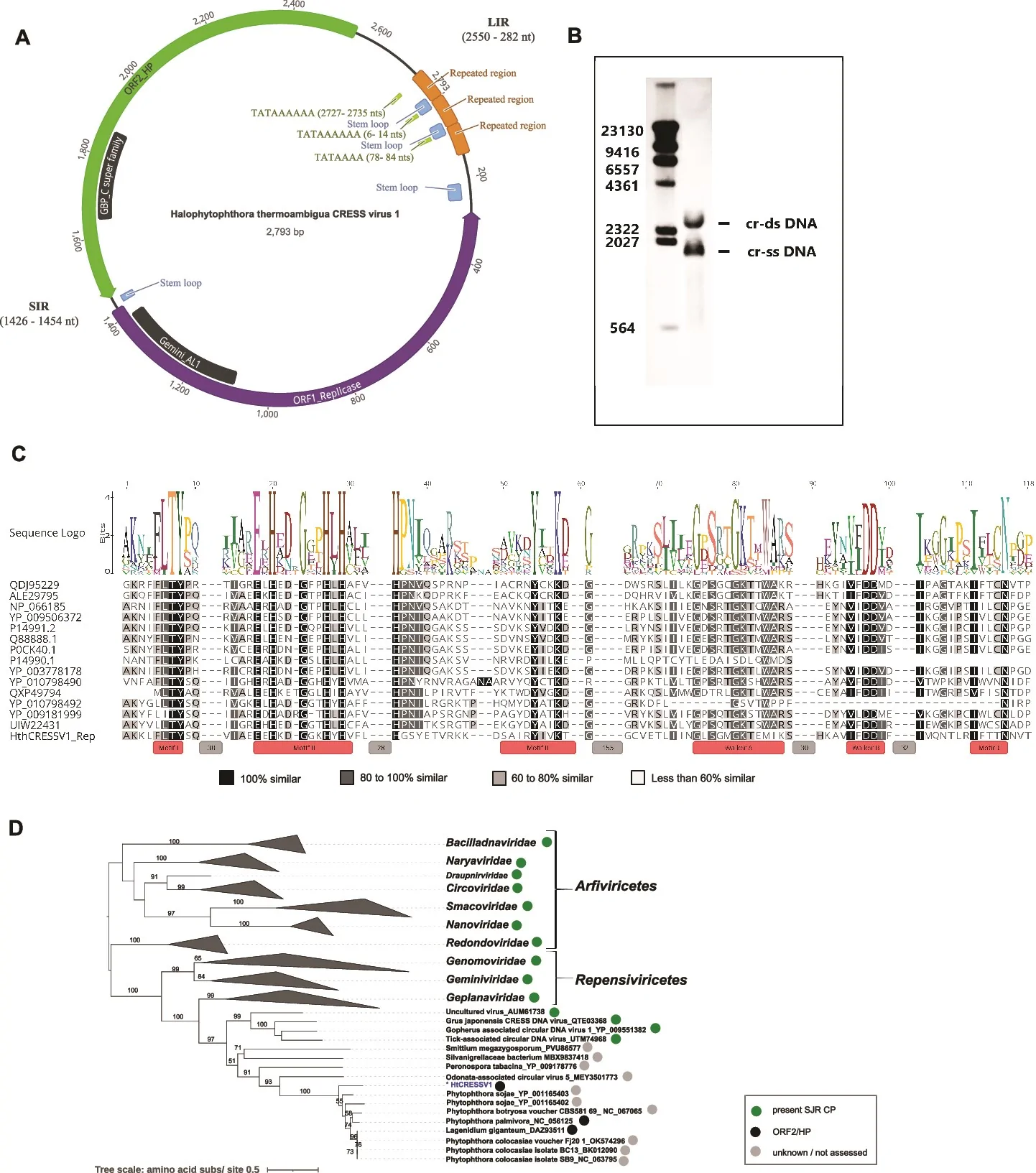
Genome organization and rep motif architecture of HthCRESSV1. (A) Schematic representation of the circular genome of HthCRESSV1 showing the two major ORFs encoding the replication-associated protein (Rep) and the hypothetical protein (HP), as well as the large (LIR) and small (SIR) intergenic regions. Predicted stem–loop structures and repeated elements are indicated. (B) Southern blot analysis of HthCRESSV1. M, digoxigenin-labelled DNA size marker (λ DNA digested with HindIII). The viral DNA forms are indicated as follows: double-stranded (ds) DNA; cr, circular single-stranded (ss) DNA. Both forms migrate faster than the linear full-length genome (~2.7 kb, determined by inverse PCR), consistent with their circular/single-stranded conformations rather than a difference in sequence length. (C) Comparison of Rep motif composition across representative members within the order Geplafuvirales and HthCRESSV1, including rolling-circle replication motifs (I–III) and helicase motifs (Walker A, Walker B, and motif C). Geplanaviridae: QDJ95229 (Capybara virus 11), NP_066185 (Horseradish curly top virus); Geminiviridae: ALE29795 (Lake Sarah-associated circular virus 45); YP_009506372 (Abutilon golden mosaic Yucatan virus), P14991.2 (Beet curly top virus), Q88888.1 (Tomato pseudo-curly top virus), P0CK40.1 (Bean golden yellow mosaic virus), YP_003778178 (Turnip curly top virus); Genomoviridae: YP_010798490 (*Zizania latifolia* genomoviridae), YP_010798492 (Artemisia carvifolia genomoviridae), YP_009181999 (Soybean associated gemycircularvirus 1), UIW22431 (Sclerotinia sclerotiorum hypovirulence associated DNA virus 1), QXP49794 (Botrytis gemydayirivirus 2). Numbers in grey boxes represent number of nucleotides deleted in the alignment. (D) Randomized Axelerated ML tree (RAxML) depicting the phylogenetic relationships of the predicted Replicase. Nodes are labelled with bootstrap support values ≥50%. Branch lengths are scaled to the expected underlying number of substitutions per site. Some of the classified families and genera are collapsed to simplify the tree. Family classification and the corresponding GenBank accession numbers are shown next to the virus names. Scale bars represent expected changes per site per branch. Presence of a capsid protein with a single jelly-roll fold (SJR CP), and an ORF2-encoded hypothetical protein (HP) as described in this study, are indicated by green and black circles, respectively. Grey circles indicate features that are unknown or could not be confidently assigned.

To identify the *Halophytophthora* strains infected with the putative ssDNA virus, total nucleic acids were extracted from the eight strains originally subjected to transcriptomic analysis, and virus identification was performed by PCR amplification using a pair of specific primers designed based on the contig sequence (Table S2). The positive signal was obtained exclusively with the DNA extracted from *H. thermoambigua* BD651, the ex-type strain of the species isolated from brackish water in Parque Natural da Ria Formosa, Santa Luzia, Tavira (Portugal). To obtain the complete genome of the putative ssDNA virus, DNA extracted from isolate BD651 was amplified by PCR using abutting primers (Table S2). After DNA purification, cloning, and sequencing, a complete circular genome of 2793 nt was obtained (Figs 1A and S1) and denoted Halophytophthora thermoambigua circular Rep-encoding single-stranded DNA virus 1 (HtCRESSV1) (GenBank accession number PV883009, SRA Bioproject PRJNA1437999). Although our data do not support a multipartite genome, we cannot formally rule out that a highly divergent or very low-abundance additional segment(s) escaped detection by homology-based searches.

To further confirm the presence of HtCRESSV1 in *H. thermoambigua* and to assess the existence of the single-stranded and double-stranded replicative intermediates expected of viruses that replicate by rolling circle mechanism (as suggested by the presence of the *rep* gene), purified DNA samples obtained from mycelia were subjected to Southern hybridization with HtCRESSV1-specific probes. Two HtCRESSV1-specific bands were detected: one displayed faster mobility than the 2027 bp marker of λDNA digested with HindIII, whereas the second one displayed slower mobility and exceeded 2322 bp (Fig. 1B). The distinct nature and size of the faster-migrating band are consistent with a circular ssDNA genome of ~2 kb, whereas the slower-migrating band is compatible with a dsDNA-replicative intermediate; both are essentially full-length relative to the 2793 bp genome. Their difference in electrophoretic mobility is most parsimoniously explained by a difference in conformation, a circular single-stranded genome (faster-migrating band) *versus* a circular, relaxed double-stranded replicative form (slower-migrating band), rather than by a difference in sequence length. A defective, deletion-bearing genome would instead be expected to migrate as a distinctly shorter band, which we do not observe, a pattern analogous to that reported for the ssDNA/dsDNA-replicative forms of Sclerotinia sclerotiorum hypovirulence-associated DNA virus 1 (Yu et al. 2010). The presence of two defined viral DNA forms, rather than a heterogeneous smear indicative of degradation, is consistent with the replication of the viral genome as an episomal, likely circular molecule.

### 3.2. Description of the genomic features of HthCRESSV1

The complete HtCRESSV1 genome contains two open reading frames (ORFs), both oriented in the same direction (Fig. 1A). ORF1 encodes the putative Rep of 380 aa (estimated 44.66 kDa), with the domain organization typical of members in the phylum *Cressdnaviricota* (Kazlauskas et al. 2019), whereas ORF2 encodes a hypothetical protein (HP) of 363 aa (estimated 43.19 kDa). The two ORFs are separated by two intergenic regions. The smaller intergenic region (SIR) comprises a 29-nt AT-rich sequence located between the stop codon of the HP and the start codon of the Rep and has the potential to form a stable secondary structure (Figs 1A and S2). The larger intergenic region (LIR) of 529 nt separates the 3′ end of Rep and the 5′ end of HP. A putative TATA-like promoter motif (TATATATATTACTA) is present at the 5′ end of the LIR, suggesting it may function as the transcriptional start site for the downstream HP gene. Notably, LIR features several tandemly repeated 72-nt units (TAGATTATAAAAAACCACAAGAAAGAATTGGTTTTTAGTTAAAAAGTTGGTTTTTAGGTTTTTTATATGGTT), encompassing fragments capable of forming stable stem-loop structures (Figs 1A and S2). The 3′ end of LIR includes a predicted stable hairpin with a 9-nucleotide loop, which could serve as the origin of replication, where the viral Rep initiates the rolling circle replication by introducing a nick. Notably, the sequence of the putative nonanucleotide, TGGCAGTGT, present at the apex of the hairpin is different from those predicted for other well studied cressdnaviricots, such as geminiviruses, genomoviruses, smacoviruses, or circoviruses (Varsani and Krupovic 2017, 2018, Fiallo-Olivé et al. 2021). Thus, the overall architecture of the LIR, including its AT-richness, promoter-like motifs, and stem-loop structure support its potential dual role in initiating both replication and transcription.

### 3.3. Comparative analysis of Rep and HP sequences

Multiple Rep sequence alignment demonstrated conservation of canonical rolling-circle replication (RCR) motifs I–III and helicase-associated Walker A, Walker B, and Motif C regions, confirming that HthCRESSV1 encodes a putatively functional rolling-circle Rep protein (Fig. 1C).

Maximum likelihood phylogenetic analysis placed HthCRESSV1 within a clade of unclassified viruses assigned to the order *Repensiviricetes*. This assemblage of unclassified viruses is more closely related to the family *Geplanaviridae* than to *Genomoviridae* or *Geminiviridae* (Fig. 1D), highlighting its distinct evolutionary position within *Cressdnaviricota*. In addition to viral homologues, BLAST searches identified Rep-like sequences embedded in the mitochondrial genomes of several oomycetes, including *L. giganteum*, *P. sojae*, and *Peronospora tabacina* (Table S3). These sequences cluster with HthCRESSV1 in the phylogenetic tree, indicating shared evolutionary ancestry between the viral Rep and mitochondrial Rep-like elements in oomycetes.

The BLASTn search revealed high nucleotide identity (76.88%) between HtCRESSV1 and an unannotated region of the mitochondrial genome of *P. palmivora* (culture ICMP:17709; NC_056125.1; 80% query coverage, *E*-value = 0.0). Similar matches were detected in mitochondrial sequences of other oomycetes, including *P. colocasiae*, *P. botryosa*, and *Pseudoperonospora cubensis* (Table S4). These homologous regions included the coding regions of both HtCRESSV1 Rep (Table S4) and HP (Table S5). Alignment of the HtCRESSV1 HP with the homologous mitochondrial sequences revealed strong aa conservation across several regions (Fig. S4).

**Figure 2:**
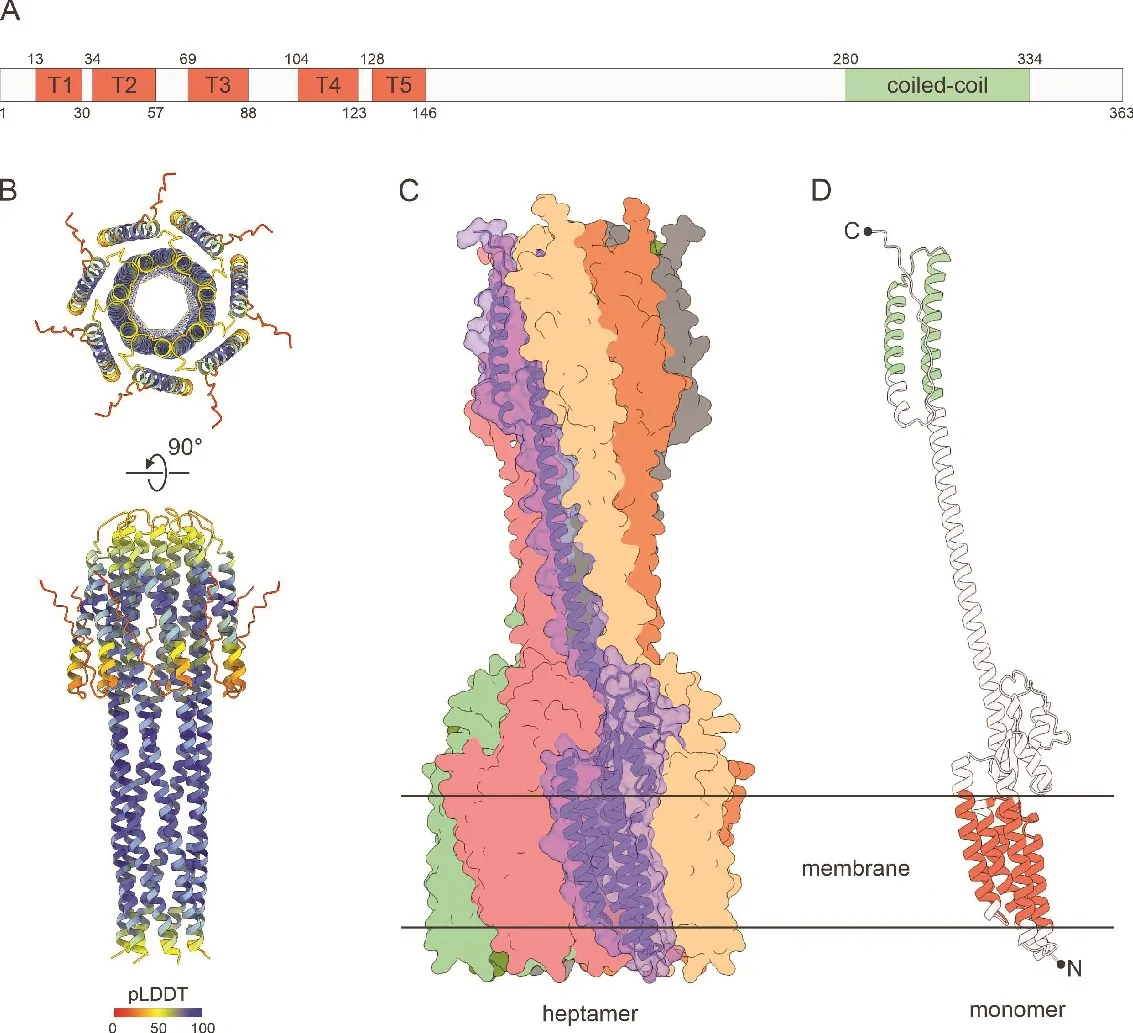
Structural modelling of the ORF2-encoded hypothetical protein. (A) Sequence features of the HthCRESSV1 hypothetical protein (HP). The sequence is represented by a rectangle, with the positions of the five transmembrane domains (T1–5) and the coiled-coil region indicated. (B) Structural model of the heptamer of the HP ectodomain generated with AlphaFold3. The model is coloured based on the predicted local distance difference test (pLDDT) score, a per-residue measure of local confidence, from red to blue (the colour scale is provided at the bottom of the figure). (C) Structural model of a heptamer of the full-length HP. surface representation is shown, with each subunit coloured differently. For one subunit, the surface is set to be semitransparent to show the secondary structure elements. (D) A monomer of the full-length HP. One of the subunits modelled in the context of a heptamer is shown separately and coloured to depict the membrane-spanning domains and the coiled-coil region, as indicated in panel (A).

In all characterized cressdnaviricots, the second of the two major ORFs encodes a capsid protein with the jelly-roll fold. Unexpectedly, sequence analysis showed that HP encoded by ORF2 does not have the potential to adopt the jelly-roll fold and instead is likely to be a membrane protein. The protein is predicted to be predominantly α-helical (70.2%), with β-stranded regions and unstructured coils contributing 6.6% and 23.2%, respectively. Five transmembrane domains were predicted in the N-terminus of the protein, whereas ~50 C-terminus proximal amino acids were predicted to form a coiled coil (Fig. 2A). These features are unprecedented among proteins encoded by cressdnaviricots. Indeed, BlastP searches queried with the HP sequence against viral databases at NCBI and IMG/VR yielded no significant hits. Homologues of HP were exclusively encoded within the above-mentioned putative EVEs identified in oomycete mitochondria, suggesting specific association with HthCRESSV1-like viruses. To gain further insight into the topology and structure of HP, we attempted to model its structure using AlphaFold3 (Zimmermann et al. 2018). Given that AlphFold3 does not explicitly model membrane proteins in their natural membrane environment, we opted for modelling only the HP ectodomain first, i.e. the domain that follows the transmembrane helices and is predicted to extend away from the membrane. To determine the likely oligomeric state of HP, we modelled multimers with progressively increasing oligomeric states from monomer to decamer and compared their predicted template modelling (pTM) and interface predicted template modelling (ipTM) scores (Table S6). The pTM and ipTM scores gradually improved from monomer to heptamer (best scores: ipTM = 0.77, pTM = 0.77), and started to decrease again for higher oligomeric states, suggesting that HP forms heptamers (Figs 2B and S4). In the modelled heptamer structure, the subunits arranged into an elongated tube-like structure, where the central α-helix formed contacts with the neighbouring subunits. The predicted coiled-coil region was predicted to fold on itself, forming intramolecular contacts at the distal part of the ectodomain. We next modelled oligomers of the full-length HP. As in the case of the ectodomain, the highest pTM and ipTM scores were obtained for the hexameric and heptameric states, although the overall scores were considerably lower than for the ectodomain (Table S6). Nonetheless, the ectodomain model could be superposed with the full-length model (root-mean-square deviation [RMSD] of 2 Å over 132 residues), supporting the relevance of the full-length heptameric model (Fig. 2C and D). Thus, structural modelling suggests that HP forms an extended, membrane embedded spike-like complex. Structure-based searches with the monomer or heptamer against structure databases using DALI (Holm 2022) and FoldSeek (van Kempen et al. 2024) did not reveal structural homologues, further emphasizing the distinctiveness of the HP compared to the previously characterized cellular and viral proteins.

### 3.4. Visualization of possible virus-like particles

**Figure 3:**
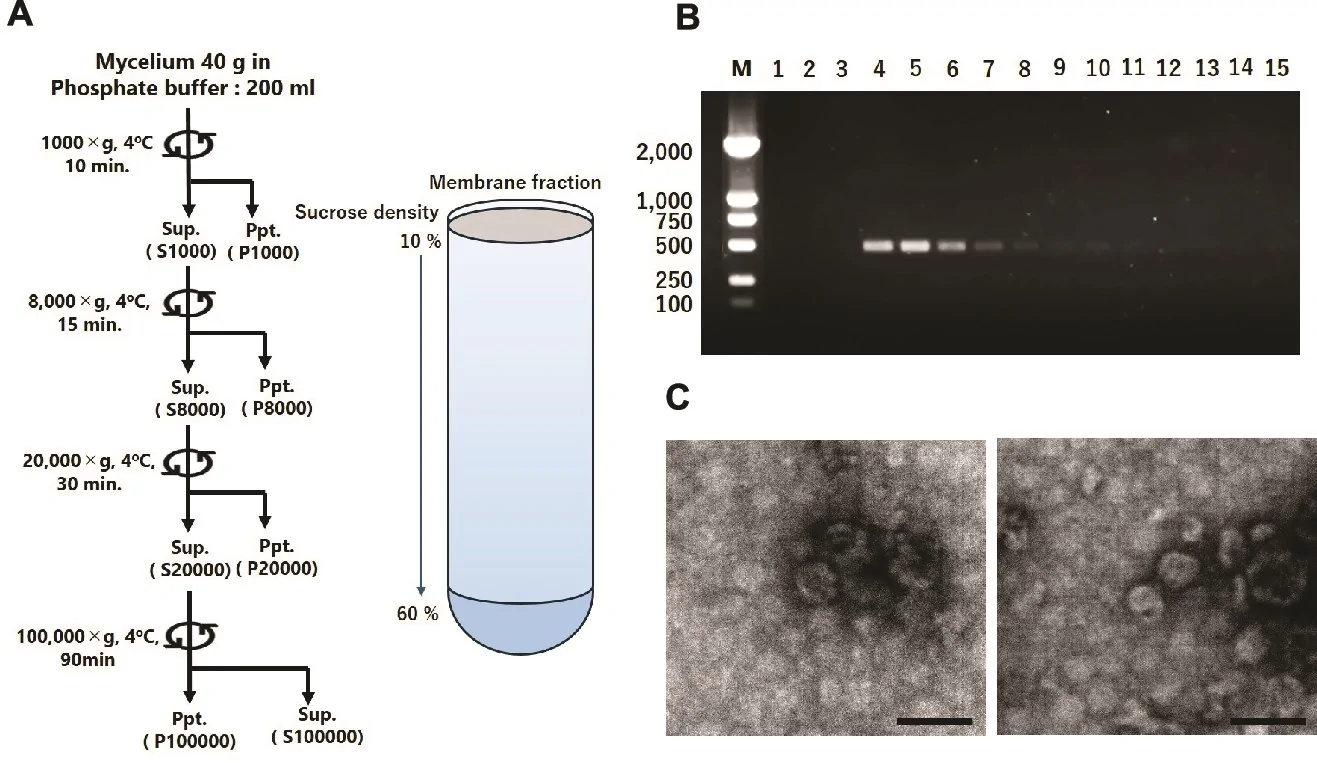
Purification attempts of HthCRESSV1 virus-like particles (VLPs). (A) Overview of virus-associated particle purification from strain BD651. Left: Mycelium homogenized in liquid nitrogen was suspended in isotonic solution and subjected to differential centrifugation at 1000, 8000, 20 000, and 100 000 *g*. Pellets (Ppt) and supernatants (Sup) were collected at each step and analysed. Right: The P100 000 pellet was subjected to continuous sucrose density gradient centrifugation (10%–60% w/v). Following ultracentrifugation, 2 ml aliquots were collected from the top, yielding 15 fractions. (B) PCR detection of HthCRESSV1 in the 15 fractions obtained by density gradient centrifugation. M, DNA size marker. (C) Transmission electron microscopy (TEM) of pooled fractions 4 and 5. Samples were negatively stained with 1% uranyl acetate. Scale bars, 50 nm.

The presence of viral particles has been demonstrated for a number of fungal cressdnaviricots. To verify whether HthCRESSV1 is capable of forming VLPs, despite lacking the gene for canonical capsid protein, we attempted to purify VLPs using standard purification protocols and visualized them by TEM (Fig. 3A). Following fractionation of the putative VLP preparation on the continuous sucrose density gradient, 15 fractions were collected and analysed for the presence of HthCRESSV1 genome using PCR with specific primers. HthCRESSV1 genomic DNA was abundant in fractions 4 and 5 (15%–20% sucrose) (Fig. 3B). TEM examination of these fractions revealed numerous spherical, pleomorphic particles that co-fractionated with HthCRESSV1 DNA. Consistent with the absence of a canonical capsid protein gene in the HthCRESSV1 genome, no icosahedral particles were observed (Fig. 3C). In the absence of biochemical characterization of the VLPs, we refrain from referring to the observed virus-associated particles as *bona fide* HthCRESSV1 virions. Further infectivity assays and more detailed ultrastructural characterization of the particles will be required for establishing their provenance and role during infection.

### 3.5. Analysis of HtCRESSV1 mitochondrial localization

**Figure 4:**
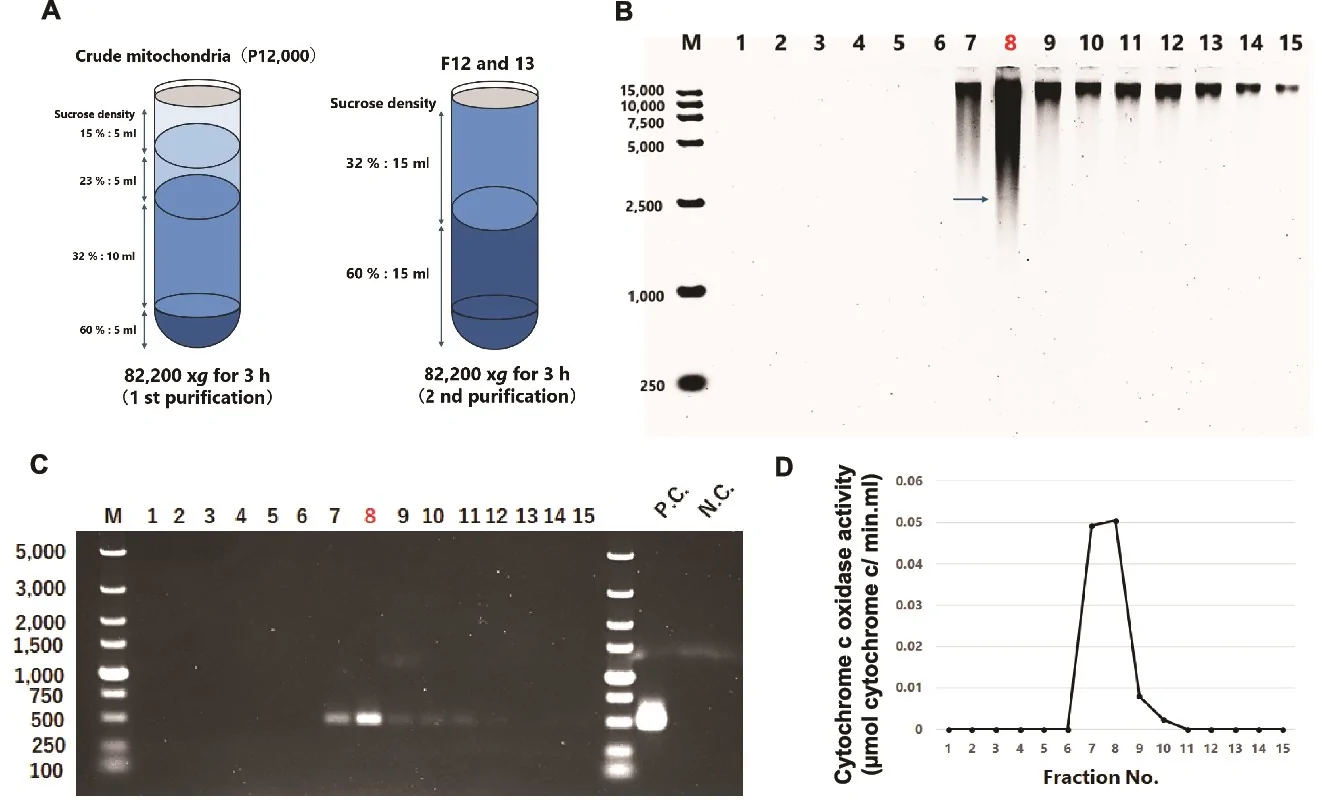
Mitochondrial localization of HthCRESSV1. (A) The P12 000 pellet was subjected to discontinuous sucrose density gradient centrifugation (15%, 23%, 32%, and 60% w/v). For the first purification, fractions collected at the 32%/60% interface (approximately fractions 12–13 from the top) were pooled and subjected to a second round of purification. Fractions (2 ml each) were collected from the top of the gradient. Total nucleic acids were extracted, ethanol-precipitated, and resuspended in TE buffer containing 1 mg ml^−1^ RNase A. (B) Agarose gel electrophoresis of total nucleic acids from the second mitochondrial purification fractions. Total nucleic acid samples extracted from each fraction were subjected to agarose gel electrophoresis from left (lane 1; 10%) to right (lane 15; 60%). The arrow indicates the HtCRESSV1 band. The image is shown with inverted contrast. M, DNA ladder (C) and (D) PCR specific for HthCRESSV1 detection and measurement of Cytochrome c oxidase (COX) activity for the second mitochondrial purification fractions. (C) HthCRESSV1-specific PCR of total nucleic acid samples extracted from each fraction. M, DNA ladder, P.C., positive control; N.C., negative control (water). (D) Cytochrome c oxidase (COX) activity in fractions from the second purification. COX activity was measured using 100 μl from each fraction, combined with 950 μl of assay buffer and 50 μl of substrate solution (reduced cytochrome c) for a total volume of 1 100 μl. Absorbance at 550 nm was measured for 1 min, and the decrease in absorbance (ΔA) was recorded. Activity was calculated in units of μmol cytochrome c/min/ml using ΔA/min and the molar extinction coefficient difference of cytochrome c (21.8 mM^−1^ cm^−1^).

Exclusive association of HtCRESSV1-like sequences with oomycete mitochondria prompted us to verify the mitochondrial localization of HtCRESSV1. To this end, the crude mitochondrial fraction (P12 000) obtained by cellular fractionation was subjected to two-step sucrose density gradient centrifugation. Following centrifugation, 15 fractions (F1–F15) containing purified mitochondria were collected (Fig. 4A), total nucleic acids were extracted from each fraction and analysed by PCR with HtCRESSV1-specific primers.

Agarose gel electrophoresis analysis of the total nucleic acids extracted from the 15 fractions revealed that fraction F8 contained the highest concentration of high-molecular-weight DNA, likely corresponding to the mitochondrial DNA. Notably, this fraction also contained a faint band of ~2500 bp in size, matching the expected size of the viral genome (Fig. 4B). Consistently, semi-quantitative PCR analysis showed that HtCRESSV1 genome was enriched in fraction F8 (Fig. 4C).

To evaluate the distribution of mitochondria in the gradient fractions, cytochrome oxidase (COX) activity, a mitochondrial marker, was measured in all 15 fractions. Strong COX activity was detected in fractions F7 and F8 (0.05 μmol cytochrome c/min/ml) indicating the presence of intact mitochondria at high concentrations in these fractions (Fig. 4D), suggesting that HtCRESSV1 localizes within mitochondria.

### 3.6. HtCRESSV1 is transmitted *via* zoospores

**Figure 5:**
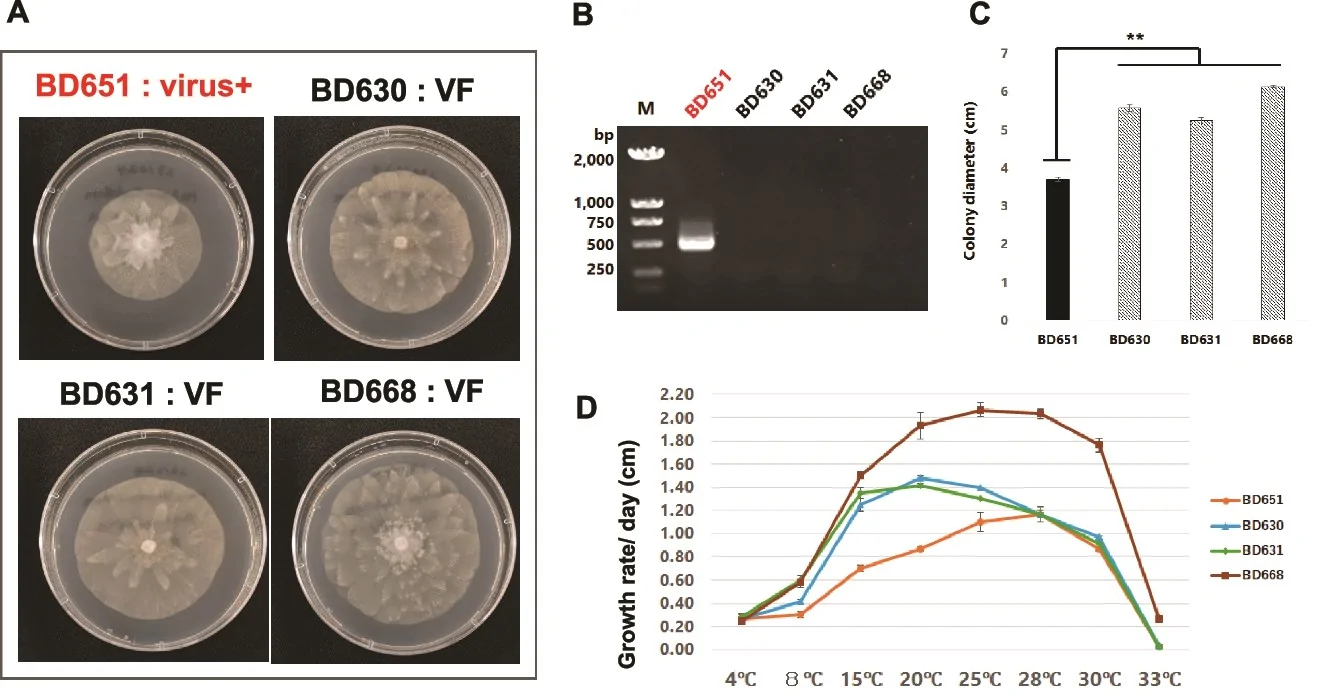
Biological effects of HthCRESSV1. (A–C) Comparison of growth between virus-infected (BD651) and virus-free *H. thermoambigua* strains (BD630, BD631, BD668). (A) Colonies grown on s. V8A medium for 3 days. From left to right: BD651 (virus-infected), BD630 (virus-free), BD631 (virus-free), BD668 (virus-free). (B) PCR detection of HthCRESSV1 in each strain. M, DNA size marker. (C) Colony diameters after 3 days of growth on s. V8A medium. Black bar represents the virus-infected strain BD651; hatched bars represent virus-free strains. The virus-infected strain showed significantly reduced growth compared to virus-free strains (*P < .01). (D) Comparison of growth rates of BD651, BD630, BD631, and BD668 at different temperatures (4°C, 8°C, 15°C, 20°C, 25°C, 28°C, 30°C, and 33°C). Growth rates were calculated as colony diameter per day on s. V8A medium. Symbols: circles, BD651 (virus-infected); triangles, BD630 (virus-free); diamonds, BD631 (virus-free); squares, BD668 (virus-free). Note that the optimal growth temperature was 28°C for BD651, 20°C for BD630 and BD631, and 25°C for BD668.

To verify whether HtCRESSV1 can be transmitted *via* zoospores we analysed single-lesions formed by zoospore-derived isolates. All 25 strains derived from zoospores isolated from single lesions formed on cork oak leaves retained HtCRESSV1 infection (Fig. 5A), indicating 100% zoospore transmission rate of the virus.

To obtain virus-free strains, treatment with the antiviral agents, ribavirin and cycloheximide, a method frequently employed for mycovirus clearance (Uchida et al. 2021), was performed. However, no virus-free strains were successfully isolated among the 20 oomycete strains regenerated from protoplasts (Fig. S5). Similarly unsuccessful was the attempt to obtain virus-free strains by single-zoospore isolation, an approach previously used to cure fungal hosts from the cressdnaviricot Diaporthe sojae circular DNA virus 1 (Wang et al. 2024).

### 3.7. Evaluation of host impact

To investigate whether HtCRESSV1 infection affects the growth of the host oomycete, similar to what has been reported during the SsHADV-1 infection of the fungus *S. sclerotiorum*, we compared the growth of naturally virus-infected strain BD651 and noninfected strains BD630, BD631, and BD668. First, mycelial colonies were compared after 3 days of incubation at 25°C on sV8A medium (prepared with 50% seawater). The colony diameter of the virus-infected strain BD651 was significantly smaller compared to the virus-free strains (Fig. 5B and C). The virus-infected strain also showed slightly higher hyphal density consistent with slower growth.

Next, we compared the daily hyphal extension rates (cm/day) at temperatures ranging from 4°C to 33°C. The optimal growth temperatures (temperatures with the highest hyphal extension rates) for BD651, BD630, BD631, and BD668 were 28°C (1.17 cm/day), 20°C (1.48 cm/day), 20°C (1.42 cm/day), and 25°C (2.07 cm/day), respectively (Fig. 5D). Thus, virus-infected strain BD651 had the highest optimal growth temperature. Notably, however, the virus-infected strain BD651 was characterized by slower growth rate compared to the noninfected strains at lower (suboptimal) temperatures in the range of 4°C–25°C. These results suggest that HtCRESSV1 infection negatively impacts growth rates of the oomycete across a wide temperature range, most likely reducing its ability to colonize plant tissues.

## 4. Discussion

In this study, we identified a novel virus with circular ssDNA genome, HthCRESSV1, associated with the marine oomycete *H. thermoambigua*. Although viruses with linear ssDNA genomes classified in the recently created family *Oomyviridae* (phylum *Cressdnaviricota*, class *Arfiviricetes*, order *Lineavirales*) have been predicted to infect oomycetes based on the presence of related EVEs in the genomes of diverse oomycetes species (Sabanadzovic et al. 2025), to the best of our knowledge, HthCRESSV1 represents the first experimentally characterized cressdnaviricot infecting an oomycete. Indeed, all viruses infecting marine fungi or oomycetes isolated up to now have RNA genomes (Nerva et al. 2016, 2019, Botella et al. 2020, Botella and Jung 2021). This is in line with the fact that most known mycoviruses, regardless of the ecosystem they inhabit, have dsRNA or ssRNA genomes, whereas ssDNA mycoviruses are relatively rare (Kondo et al. 2022). Notably, HthCRESSV1 is unrelated to viruses of the family *Oomyviridae* (Sabanadzovic et al. 2025), which have linear genomes, encode a typical jelly-roll capsid protein, and based on the Rep phylogeny are placed within the class *Arfiviricetes*. By contrast, Rep phylogeny firmly places HthCRESSV1-like viruses within the class *Repensiviricetes*, as a separate family, which we propose naming ‘*Mitodnaviridae*’.

Arguably the most unexpected feature of HthCRESSV1 is its association with mitochondria. Detection of HthCRESSV1-like EVEs in oomycete species from different genera suggests that the association with mitochondria is lasting, rather than transient. The only other previously known group of viruses associated with mitochondria are ssRNA viruses of the family *Mitoviridae*, which replicate in mitochondria of filamentous fungi (Polashock and Hillman 1994, Polashock et al. 1997). Phylogenetic analysis of the Rep positions HthCRESSV1 at the base of the clade including related EVEs, suggesting that the latter derive from the integration HthCRESSV1-like viruses into mitochondrial genomes. Notably, Southern blot analysis indicated that the host strain does not carry an integrated copy of HthCRESSV1, suggesting that integration into the mitochondrial genome is not an obligatory step of virus replication. HthCRESSV1 and related EVEs are most closely related to unclassified and uncultivated viruses, some of which encode typical jelly-roll capsid proteins, suggesting that HthCRESSV1 lineage has evolved from *bona fide* cressdnaviricots following the replacement of the ancestral capsid protein gene with the one encoding the membrane-associated ORF2 protein, an α-helical membrane protein with five N-terminal transmembrane domains and a C-terminal coiled-coil region. Structural modelling suggests that this protein can form multimeric complexes within membranes, likely as heptamers. Homologues of ORF2 were not detected in known members of the phylum *Cressdnaviricota* but were identified in several oomycete mitochondrial genomes (Martin et al. 2007, Derevnina et al. 2015, Winkworth et al. 2020, 2022, Morgan and Tartar 2023) suggesting an evolutionary relationship with mobile mitochondrial elements or integrated viral fragments. These homologues often occur without adjacent *rep* genes, implying independent acquisition through horizontal transfer or secondary loss of *rep*. The tight association of the HthCRESSV1 genome with mitochondrial DNA, coupled with the absence of nuclear integration, supports the hypothesis that replication occurs exclusively within mitochondria. This is further reinforced by TEM analysis, which revealed vesicle-like structures potentially associated with viral replication.

Functionally, ORF2 likely contributes to membrane dynamics. If the viral genome is encapsulated within membrane-bound vesicles, ORF2 may mediate vesicle formation, budding, or membrane penetration events that facilitate virus spread, replication, genome packaging, or evasion of mitochondrial antiviral defences (Miller and Krijnse-Locker 2008, Hsu et al. 2010). Alternatively, if the virus remains confined within the mitochondrial compartment, like in the case of the RNA mitoviruses, ORF2 may remodel inner membrane structures such as cristae or mitovesicles to create specialized niches for replication (Kopek et al. 2007, den Boon and Ahlquist 2010). The presence of a C-terminal coiled-coil domain supports a role in oligomerization or scaffolding, common features of membrane-associated structural or assembly proteins (Burkhard et al. 2001, Lupas and Bassler 2017). Given its distinctive combination of features and phylogenetically isolated position, ORF2 likely represents a novel membrane-active viral factor, possibly acquired through horizontal transfer from a mitochondrial mobile element or host organellar DNA.

Overall, the evolutionary history of HthCRESSV1 is consistent with the current views on the cressdnaviricot evolution, which involves recurrent recombination between plasmid-like replicons leading to repeated acquisition, exchange, and loss of capsid protein genes (Koonin et al. 2015, Kazlauskas et al. 2018, Desingu and Nagarajan 2022, Krupovic and Koonin 2026). In this framework, the Rep phylogeny firmly roots HthCRESSV1 within *Repensiviricetes*, attesting to its viral origin. By contrast, replacement of the canonical jelly-roll capsid protein gene with HP in the ancestor of the proposed family ‘*Mitodnaviridae*’ could be associated with the relocation to mitochondria as the replicative compartment. In a sense, this transition might be considered as return to the roots, considering that cressdnaviricots are believed to have evolved from bacterial plasmids (Kazlauskas et al. 2019), whereas mitochondria represent domesticated alphaproteobacterial endosymbionts.

From a virological perspective, most DNA viruses, with the notable exception of certain large dsDNA viruses from the phylum *Nucleocytoviricota*, replicate in the nucleus. It remains unclear whether minimal cressdnaviricot viruses, such as HthCRESSV1, possess the capacity to actively cross the mitochondrial double membrane. Information on viruses that directly interact with mitochondria is extremely limited. For example, mitovirus-derived sequences have been identified in both the nuclear and mitochondrial genomes of several vascular plants (Hong et al. 1998, Xu et al. 2014, Bruenn et al. 2015, Nibert et al. 2018). Among infectious DNA viruses, a reverse-transcribing hepatitis B virus (HBV) not only integrates into the host nuclear genome but has also been shown to deliver HBV RNA into mitochondria of hepatocellular carcinoma cells, where it may participate in mtDNA integration (Giosa et al. 2023). This process is mediated by polynucleotide phosphorylase (PNPase), which facilitates RNA transport into mitochondria. Given the RNA specificity of PNPase, its involvement in the translocation of the DNA genome of HthCRESSV1 into mitochondria is unlikely. Further studies using immunoelectron microscopy or other imaging approaches will be required to determine whether HthCRESSV1 localizes to the mitochondrial matrix or the intermembrane space.

Consistent with this proposed mitochondrial association, indirect evidence supporting the interaction between HthCRESSV1 and mitochondria includes its highly efficient vertical transmission *via* zoospores and variation in host growth across temperatures. The species epithet of *H. thermoambigua* reflects its ambiguous optimal growth temperature (Maia et al. 2022); comparative growth assays showed that, compared to the virus-free strains, the virus-infected strain BD651 exhibited a reduced growth overall while maintaining relatively similar growth at higher temperatures. Mitochondrial genomes have been implicated in thermal adaptation in several organisms, including *P. infestans*, *Drosophila melanogaster*, and *Saccharomyces cerevisiae* (Camus et al. 2017, Li et al. 2019, Shen et al. 2022). Although HthCRESSV1 is not integrated into mtDNA, its association with mitochondria may influence organelle function and contribute to reduced growth rates and elevated optimal growth temperature. In this context, mitochondrial RNA mitoviruses provide a useful comparison. Although evolutionarily unrelated to HthCRESSV1 and typically associated with cryptic infections, they share a common intracellular niche and are transmitted vertically through spores very efficiently (Polashock et al. 1997, Čermáková et al. 2017, Schoebel et al. 2017). In some cases, mitoviruses have been linked to altered host phenotypes; for example, infection by Sclerotinia sclerotiorum mitovirus 1 (SsMV1) in *S. sclerotiorum* is associated with reduced growth and hypovirulence, alongside mitochondrial abnormalities (Xu et al. 2014).

Our attempts to eliminate HthCRESSV1 using ribavirin were unsuccessful, and the virus remained stably transmitted through zoospores. Similar persistence has been reported for other cressdnaviricots infecting fungi (Wang et al. 2024). Notably, successful re-infection has been demonstrated for certain viruses such as SsHADV-1 using purified virions or infectious clones (Yu et al. 2010, Wang et al. 2024). Developing a re-infection system for HthCRESSV1, potentially through mitochondrial transfection approaches, will be essential to clarify its biological effects on the host.

## 5. Conclusion

HthCRESSV1 represents a circular DNA virus with unique genomic features and a close association with oomycete mitochondria. Its genome encodes a divergent Rep protein and a membrane-associated HP conserved in mitogenomes, suggesting prolonged interaction with the mitochondrial compartment. The absence of recognizable capsid genes and the presence of repetitive regulatory elements point to a separated evolutionary trajectory within cressdnaviricots. Together, these findings expand current views of CRESS virus diversity and highlight mitochondria as a potential niche for circular DNA replicons in eukaryotes.

## Supplementary Material

Supplementary_Files_veag047

## Data Availability

The genome sequence of Halophytophthora thermoambigua circular Rep-encoding single-stranded DNA virus 1 (HtCRESSV1) is available in GenBank under accession number PV883009. The raw sequencing reads have been deposited in the Sequence Read Archive (SRA) under BioProject accession PRJNA1437999.
