# Supplementary material for: A single-stranded DNA virus replicates in the mitochondria of a marine oomycete
Source: Virus Evol. 2026 Jul 31;12(1):veag047. doi: 10.1093/ve/veag047 (PMC13499601; doi:10.1093/ve/veag047)
Supplement: Supplementary file 1 [file supplementary_files_veag047.pdf]

Supplementary tables

Table S1. Overview of the *Halophytophthora* isolates analyzed in this study and included in the RNA pools for total RNA sequencing.

Species	Isolate code	Pool	Site	Reference
H. thermoambigua	BD651 = CBS 147229	Yes	Parque Natural da Ria Formosa, Santa Luzia, Tavira	Maia et al. 2022
H. thermoambigua	BD630	No	Rio Séqua, Tavira	Maia et al. 2022
H. thermoambigua	BD631	No	Rio Séqua, Tavira	Maia et al. 2022
H. thermoambigua	BD668	No	Ria de Alvor, Alvor, Portimão	Maia et al. 2022
H. lusitanica	BD686 = CBS 147231	Yes	Parque Natural da Ria Formosa, Almancil, Loulé	Maia et al. 2022
H. lateralis	BD657 = CBS 147233	Yes	Parque Natural da Ria Formosa, Quelfes, Olhão	Maia et al. 2022
H. frigida	BD655 = CBS 147235	Yes	Parque Natural da Ria Formosa, Santa Luzia, Tavira	Maia et al. 2022
H. sinuata	BD656 = CBS 147237	Yes	Parque Natural da Ria Formosa, Santa Luzia, Tavira	Maia et al. 2022
H. macrosporangia	BD639 = CBS 147290	Yes	Parque Natural da Ria Formosa, Santa Luzia, Tavira	Maia et al. 2022
H. brevisporangia	BD662 = CBS 147238	Yes	Parque Natural da Ria Formosa, Quelfes, Olhão	Maia et al. 2022
H. celeris	BD885 = CBS 147240	Yes	Parque Natural da Ria Formosa, Santa Luzia, Tavira	Maia et al. 2022

Table S2. Primers used for PCR and Sanger sequencing

Primer name	Sequence (5'-3')	T_m
Halo_genomo1632V2/F	GACGACTATCCATATCTACTTGAATTTG	58.2
Halo_genomo256V2/R	AACGAGAGCTAACACTGCCATAG	60.4
Halo_genomo593V2/F	ATCTGAAATAGCTATATGTTCTTCGGATG	59.5
Halo_DNA_121 R	ACAGCAAATCCTTCAAATATACGTA	56.9
Halo_DNA_280 F	TCATTTCTGTTTTACCCATTCCAGA	58.7
Halo_DNA_536 R	AGGAGGACTAACAGCAGTAAAAGAT	59.5
Halo_DNA_780 F	CCATCCTTATGTTCTTCTTCTGCAA	59.4

Table S3. Summary of homologous sequences identified in the mitochondrial genomes of various oomycete species, based on GenBank data.

Organism/isolate	mtDNA accession number	mtDNA size (pb)	REP	ORF nt interval	Gemini_AL1 domain	Acc. Number	HP	ORF nt interval	domain	Acc. Number
*Phytophthora capsic*i isolate TaiwanPc377	BK012088	38,418	N	N	N	N	Y	6,514 - 7,627	SMC_N super family	not annotated
Phytophthora capsici isolate TaiwanPc33e	NC_063804	38,427	N	N	N	N	Y	6,514 - 7,627	SMC_N super family	not annotated
Pseudoperonospora humuli	MH923236	39,087	N	N	N	N	orf168	8,382 - 7,303	N	QLD32048.1
Pseudoperonospora cubensis	NC_027859	38,995	N	N	N	N	orf306 gene	8,779 - 7,859	N	YP_009164875.1
Phytophthora umbellatus (*Phytophthora* sp. ML-2023a)	OQ735416	39,675	N	N	N	N	Y	19,330 - 18,332	N	not annotated
Paralagenidium karlingii	NC080886	46,638	N	N	N	N	ORF22	27,753 - 29,075	N	YP_010886810
Paralagenidium karlingii	NC080886	46,638	N	N	N	N	ORF16	21,979 - 23,055	Zinc finger-containing protein; pfam10146	YP_010886807
Peronospora tabacina	NC_028331	43,225	orf374	9,988 - 11,208	Walker A and B motifs	YP_009178776	N	N	N	N
Phytophthora botryosa voucher CBS581_69_ORF 35_NC_067065	NC_067065	39,743	Y,	18,882 - 17,707	Y	not annotated	N	N	N	N
Phytophthora colocasiae voucher_Fj20_1	OK574296	41,838	Y	19,604 - 18,456	Y	not annotated	Y, shorter	20,649 - 19,918	N	not annotated
Phytophthora colocasiae isolate BC13	BK012090	41,367	Y	8,451 - 9,599	Y	not annotated	N	N	N	N
Phytophthora colocasiae isolate SB9	NC_063795	41,297	Y	8,381 - 9,529	Y	not annotated	N	N	N	N
Phytophthora palmivora	NC_056125	40,799	Y	8,338 - 9,549	Y	not annotated	Y	7,200 - 8,363	SMC_prok_B super family	not annotated
Lagenidium giganteum strain ARSEF 373	DAKRPA010000311 *	80,403*	Y	45,029 - 45,667	Y	DAZ93511	Y	46,469 - 47,064	Coiled-coil domain-containing protein 158; CCDC158	DAZ93512
Phytophthora sojae	NC_009385	42,977	orf98	29,048 - 29,344	Y	YP_001165402	N	N	N	N
Phytophthora sojae	NC_009385	42,977	orf112a	29,328 - 29,666	N	YP_001165403	N	N	N	N

Y, Yes, Present; N, No, absent; **Lagenidium giganteum* strain ARSEF 373 scaffold_527 mitochondrial, whole genome shotgun sequence

Table S4. Pairwise amino acid identity percentages among homologous replicases from distinct organisms.

	Phytophthora sojae hypothetical protein_YP_001165403	*Phytophthora palmivora* ORF_056125	*Phytophthora colocasiae voucher Fj20_1_ORF 31_OK574296*	*Phytophthora colocasiae* isolate SB9_ORF 10_NC_063795	*Phytophthora colocasiae* isolate BC13_ORF 21_BK012090	*Lagenidium_giganteum*_hypothetical_protein_DAZ93511	*Phytophthora botryosa* voucher CBS581_69_ORF 35_NC_067065	HtCRESSV1	*Phytophthora sojae* hypothetical protein_YP_001165402	*Peronospora tabacina* orf374_YP_009178776
Phytophthora sojae hypothetical protein_YP_001165403	0									
Phytophthora palmivora ORF_056125	43.94									
Phytophthora colocasiae voucher Fj20_1_ORF 31_OK574296	41.62	81.77								
Phytophthora colocasiae isolate SB9_ORF 10_NC_063795	41.62	82.03	99.74							
Phytophthora *colocasiae* isolate BC13_ORF 21_BK012090	41.62	82.03	99.74	100.00						
*Lagenidium_giganteum*_hypothetical_protein_DAZ93511	42.05	83.26	98.60	99.07	99.07					
Phytophthora botryosa voucher CBS581_69_ORF 35_NC_067065	41.41	80.40	79.95	79.69	79.69	80.00				
HtCRESSV1_Replicase	38.38	61.19	61.60	61.60	61.60	66.98	60.61			
Phytophthora sojae hypothetical protein_YP_001165402	2.13	30.40	33.49	33.49	33.49	2.13	31.02	27.32		
Peronospora tabacina orf374_YP_009178776	11.94	26.81	27.89	27.89	27.89	24.34	26.62	27.62	18.81	

LEGEND
>90 %
80-100 %
60-80 %
60-70 %
<60 %

Table S5. Pairwise amino acid identity percentages among homologous hypothetical proteins from distinct organisms.

	HtCRESSV1_putative_capside	*Phytophthora capsici isolate* TaiwanPc377_ORF1_BK012088	*Phytophthora capsici* isolate TaiwanPc33e ORF1_NC_063804	*Pseudoperonospora_humuli*_hypotehtical_protein_QLD32048.1	*Pseudoperonospora_cubensis*_hypothetical_protein_YP_009164875.1	*Phytophthora umbellatus*_ ORF 25_OQ735416	*Phytophthora_colocasiae*_voucher_Fj20_1_OK574296	*Phytophthora_palmivora*_hypothetical_protein_NC_056125	*Lagenidium_giganteum*_hypothetical_protein_DAZ93512	*Paralagenidium_karlingii*_YP_010886810
HtCRESSV1_putative_capside										
Phytophthora capsici isolate TaiwanPc377_ORF1_BK012088	58.967									
Phytophthora capsici isolate TaiwanPc33e ORF1_NC_063804	58.967	100								
*Pseudoperonospora_humuli*_hypotehtical_protein_QLD32048.1	53.659	55.313	55.313							
*Pseudoperonospora_cubensis*_hypothetical_protein_YP_009164875.1	56.875	58.958	58.958	69.707						
*Phytophthora umbellatus*_ ORF 25_OQ735416	58.808	59.831	59.831	60.452	59.935					
*Phytophthora_colocasiae*_voucher_Fj20_1_OK574296	40.341	43.103	43.103	42.241	43.218	49.394				
*Phytophthora_palmivora*_hypothetical_protein_NC_056125	57.48	58.594	58.594	63.021	66.049	63.003	55.65			
*Lagenidium_giganteum*_hypothetical_protein_DAZ93512	56.289	60.984	60.984	65.246	68.301	65.902	57.46	83.54		
*Paralagenidium_karlingii*_YP_010886810	21.705	17.373	17.373	20.043	21.407	21.409	17.045	16.564	20.308	
*Paralagenidium_karlingii*_YP_010886807	23.136	24.084	24.084	26.053	25.077	24.865	18.715	23.951	26.168	14.768

Table S6. Confidence scores for the structural models of HP generated using AlphaFold3.

	**Ectodomain**		**Full-length**	
**Oligomeric state**	ipTM	pTM	ipTM	pTM
**Monomer**	-	0.4	-	0.28
**Dimer**	0.08	0.26	0.22	0.29
**Trimer**	0.39	0.42	0.29	0.33
**Tetramer**	0.58	0.62	0.33	0.35
**Pentamer**	0.68	0.69	0.35	0.37
**Hexamer**	0.72	0.73	0.38	0.39
**Heptamer**	0.77	0.77	0.37	0.38
**Octamer**	0.76	0.76	0.37	0.38
**Nonamer**	0.74	0.75	0.35	0.36
**Decamer**	0.68	0.69	0.32	0.34

Amplicon sequences from Figure S1

>HthCRESSV1_Initial_Contig NODE_2192_length_979

CTTTATTTGTATAATTATTTTTTAATAAATTTGTTACATTATTTGTTGTAAAAATACGTAAAGTATTTTGCATAATTCGAATTGAATCTTTTATTATACGTATATTTGAAGGATTTGC
TGTATCTGAAATAGCTATATGTTCTTCGGATGATAATTCTTTTTCAAAAAATATATCATCAAAAATTACAGCTTTATGTTTTTGAGGATCATATTCACGTAATCCATGAATATTTCT
TATTAATAAAAATTTTATTCCTTGAGATTGTAAAAAGGATTTAATCATTTCTGTTTTACCCATTCCAGATAATCCATTTATTATTAATGTTTCTACACATCCTGTTGAATACCATTCT
TTTAAATCCTCTGGAATATTAAAATTTTTTACAGGTATAATAGAATTATTAAAATGATTTTCAATTTCTTTTTCATATCTAATTTTATCTATTTGTTCTAAATTTTTTAATACATTTGG
ACCTCCTTTTACCATTAACATTGGATTTGCTATTAAATGATCTTTTACTGCTGTTAGTCCTCCTTTTGCATGTAATTTAACTAAATATTCTTTAAAATCTAAATATAATTCTCCATCTA
AATATGGTAATTCCGGTTTTGCTTCAAATTTTCCTGTTTTTACAATATAACGTATTGCTGAATCTTTTTTTCGAACCGTTTCATAACTACCATGATATATTTGACCTTCTAATTCTAA
ATCTAAACAATTTGATCCATATAATTCAACTCGTTGATTAAATTCTAGAAAAACATGATAATGTTTTCCACCATCCTTATGTTCTTCTTCTGCAATTACATATTGTATTATTTTTGGT
ACTTTTTGTTCTAATTGTTCTAATATTTTTTGATTTACATTTTCTTGATTTTTTAGATTAATTTGTGAATAGGTTAAAAATAACTTTTTTGCTCTTAATCTAAATTGTTTATTTACTTTA
TTTTCTTTTTTCATATTTTAGTTTTTTAATTA

>Halo_genomo_Rv121.ab1_(reversed)

AACCATATAAAAAACCTAAAAACCAACTTTTTAACTAAAAACCAATTCTTTCTTGTGGTTTTTTATAATCTAAACCATATAAAAAACCTAAAAACCAACTTTTTAACTAAAAACCAA
TTCTTTCTTGTGGTTTTTTATAATCTAAATTTTTGAAAAATAAGATAAATACTATAAATAGCTTTTATCTTATTTTTCAATTTTATTTAAAACCAAATTAAAACGAGAGCTATGGCA
GTGTTAGCTCTCGTTTTAATAAATAAATACGCGTTAGTAATATATATATTAATTAATTTTAGATAAAAAATTATTATTATTTTGTTCTAATTCTTTTAATACTTTCATATTATGTTTAT
ATACATATTCTTCACGTTTTTTATTACGTATTTTTATTCGAACATCTATTTCAAAATCTTCTTTAAATAATGTTTTATTTATATTTAAAATAAAATATCTACTTAATACTGCTTTATTTG
TATAATTATTTTTTAATAAATTTGTTACATTATTTGTTGTAAAAATACGTAAAGTATTTTGCATAAT

>Halo_genomo_Fw780.ab1

TCCTGTTGAATACCATTCTTTTAAATCCTCTGGAATATTAAAATTTTTTACAGGTATAATAGAATTATTAAAATGATTTTCAATTTCTTTTTCATATCTAATTTTATCTATTTGTTCTA
AATTTTTTAATACATTTGGACCTCCTTTTACCATTAACATTGGATTTGCTATTAAATGATCTTTTACTGCTGTTAGTCCTCCTTTTGCATGTAATTTAACTAAATATTCTTTAAAATCT
AAATATAATTCTCCATCTAAATATGGTAATTCCGGTTTTGCTTCAAATTTTCCTGTTTTTACAATATAACGTATTGCTGAATCTTTTTTTCGAACCGTTTCATAACTACCATGATATA
TTTGACCTTCTAATTCTAAATCTAAACAATTTGATCCATATAATTCAACTCGTTGATTAAATTCTAGAAAAACATGATAATGTTTTCCACCATCCTTATGTTCTTCTTCTGCAATTAC
ATATTGTATTATTTTTGGTACTTTTTGTTCTAATTGTTCTAATATTTTTTGATTTACATTTTCTTGATTTTTTAGATTAATTTGTGAATAGGTTAAAAATAACTTTTTTGCTCTTAATCT
AAATTGTTTATTTACTTTATTTTCTTTTTTCATATTTTAGTTTTTTAATTAAAAAAATAATAATTATTCATCTCCATCAAATGCATAATTATTATCTATTTTTATTTTTTTTGAAGGATTT
TCATCACTTTTCATTTCTTCGAAATCAAAAGAATCTCTTCTTCTTTTTAATATCGCTTTTTCTTCTTTTAAATTTTCAATAGATTCTGAAAAATCTACTTTATTCCAAAAATGACGACT
ATCCATATCTACTTGAATTTGATGTATTTCTTTATTTAAATTATGAATTTTTTGATCATAATTCTCTAAACGTTTATCTCGTTGTTTAATTATATTTTG

>Halo_genomo_Fw280.ab1

ACTTTTTGTTCTAATTGTTCTAATATTTTTTGATTTACATTTTCTTGATTTTTTAGATTAATTTGTGAATAGGTTAAAAATAACTTTTTTGCTCTTAATCTAAATTGTTTATTTACTTTA
TTTTCTTTTTTCATATTTTAGTTTTTTAATTAAAAAAATAATAATTATTCATCTCCATCAAATGCATAATTATTATCTATTTTTATTTTTTTTGAAGGATTTTCATCACTTTTCATTTCTTC
GAAATCAAAAGAATCTCTTCTTCTTTTTAATATCGCTTTTTCTTCTTTTAAATTTTCAATAGATTCTGAAAAATCTACTTTATTCCAAAAATGACGACTATCCATATCTACTTGAATT
TGATGTATTTCTTTATTTAAATTATGAATTTTTTGATCATAATTCTCTAAACGTTTATCTCGTTGTTTAATTATATTTTGTTCTAATCTTATCTTATCTTGTTCTCTAATATATTCATCT
TTTTCTTTAATATGTTCAAATTTTTCTTTAATATGTTCAAATTTTTCAGTTTCAATCTTTTTGTTAAATTCAAACTGTTTTTGTTGAATTTGAACACTTGAATGTTGTAAATAACCTGT
GAATAAACTCCCAGCAGTTGCACTTGTTATAACTGTAATACCGTGTTTTTGCATCAAGTTTTTTACTTCATTAAAACCTATATATCGTTTTTGAATATAAAATAAAGTTTGATTTTT
ATTAAATCTTAAATTATTTATTTTTAAAATATTTTTAATTACATTATAATCTATAGTTTTACTATTATTTAAATTTTGAGGAATAAGAATGAATTTTTTGTCTATCAAATTAAATATAT
TTACTATTATTACACAGAAAAATACTAATATTGATAAACTATACGAATTATTCATTATGAGGAAGATAATGGTTAAACT

>Halo_CRESS_1632V2.ab1

ATGATTTTTTGATCATAATTCTCTAAACGTTTATCTCGTTGTTTAATTATATTTTGTTCTAATCTTATCTTATCTTGTTCTCTAATATATTCATCTTTTTCTTTAATATGTTCAAATTTTT
CTTTAATATGTTCAAATTTTTCAGTTTCAATCTTTTTGTTAAATTCAAACTGTTTTTGTTGAATTTGAACACTTGAATGTTGTAAATAACCTGTGAATAAACTCCCAGCAGTTGCAC

TTGTTATAACTGTAATACCGTGTTTTTGCATCAAGTTTTTTACTTCATTAAAACCTATATATCGTTTTTGAATATAAAATAAAGTTTGATTTTTATTAAATCTTAAATTATTTATTTTT
AAAATATTTTTAATTACATTATAATCTATAGTTTTACTATTATTTAAATTTTGAGGAATAAAAATGAATTTTTTTTCTATCAAATTAAATATATTTACTATTAATAAACAGAAAAATA
CTAATATTGATAAACTATACGAATTATTCATTATGAGGAAGATAATGTTTAAACTCCAACTTGATAATACAAATCCTGTTAAGAATACTATTAATTTTTGGTTTTTTTCATGCTCCA
CATAATTAAAATATTCAAAAATTAGTCCATATAATCCATTTTTGAATATTAAAAAATAATATAAACAAAAATTAAATAGATATGTATTTTTTATTTTATTTGTAATAAATATCCATT
GATTTATAAATAAAATTGAAATTATAATTATAATTGGATATAAATAAATCGATGGTAGTTCAAATACCACTTGATTTATTTTTATAATGATTAGATTTAAGAAAGTTATACATAAA
ATATAATTTATGTATATTTCATTAAAATTGTTTATTTTTTGTATCATTATCCAATCCTAAAAATTCTCTTATTGTATATTTAATAAATTTTATCATAATCTTTTTATCTTTCTTTTTATAT
TCAAGAGGCTAGCCTTTTACAACATTTTTATAGATATATTAAAAAGCCCTATATTTTATTTAAAACATCTTCCAAAACCCTTTTATTAAAGGTTTTCCCTAAACCATATAAAAAACC
TAAAAACCAACTTTTTAACTAAAAACC

>Halo_CRESS_256.ab1_(reversed)

GAATAAACTCCCAGCAGTTGCACTTGTTATAACTGTAATACCGTGTTTTTGCATCAAGTTTTTTACTTCATTAAAACCTATATATCGTTTTTGAATATAAAATAAAGTTTGATTTTT
ATTAAATCTTAAATTATTTATTTTTAAAATATTTTTAATTACATTATAATCTATAGTTTTACTATTATTTAAATTTTGAGGAATAAAAATGAATTTTTTTTCTATCAAATTAAATATAT
TTACTATTAATAAACAGAAAAATACTAATATTGATAAACTATACGAATTATTCATTATGAGGAAGATAATGTTTAAACTCCAACTTGATAATACAAATCCTGTTAAGAATACTATT
AATTTTTGGTTTTTTTCATGCTCCACATAATTAAAATATTCAAAAATTAGTCCATATAATCCATTTTTGAATATTAAAAAATAATATAAACAAAAATTAAATAGATATGTATTTTTT
ATTTTATTTGTAATAAATATCCATTGATTTATAAATAAAATTGAAATTATAATTATAATTGGATATAAATAAATCGATGGTAGTTCAAATACCACTTGATTTATTTTTATAATGATT
AGATTTAAGAAAGTTATACATAAAATATAATTTATGTATATTTCATTAAAATTGTTTATTTTTTGTATCATTATCCAATCCTAAAAATTCTCTTATTGTATATTTAATAAATTTTATCA
TAATCTTTTTATCTTTCTTTTTATATTCAAGAGGCTAGCCTTTTACAACATTTTTATAGATATATTAAAAAGCCCTATATTTTATTTAAAACATCTTCCAAAACCCTTTTATTAAAGGT
TTTCCCTAAACCATATAAAAAACCTAAAAACCAACTTTTTAACTAAAAACCAATTCTTTCTTGTGGTTTTTTATAATCTAAACCATATAAAAAACCTAAAAACCAACTTTTTAACTA
AAAACCAATTCTTTCTTGTGGTTTTTTATAATCTAAACCATATAAAAAACCTAAAAACCAACTTTTTAACTAAAAACCAATTCTTTCTTGTGGTTTTTTATAATCTAAATTTTTGAAA
AATAAGATAAATACTATAAATAGCTTTTATCTTA

Supplementary Figure S1

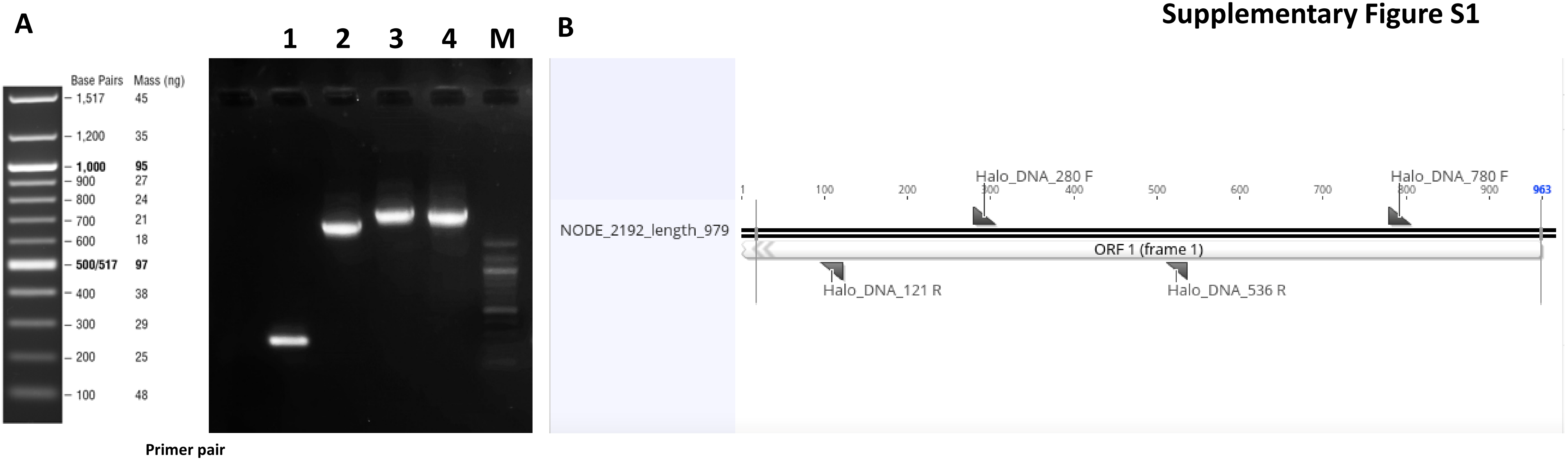

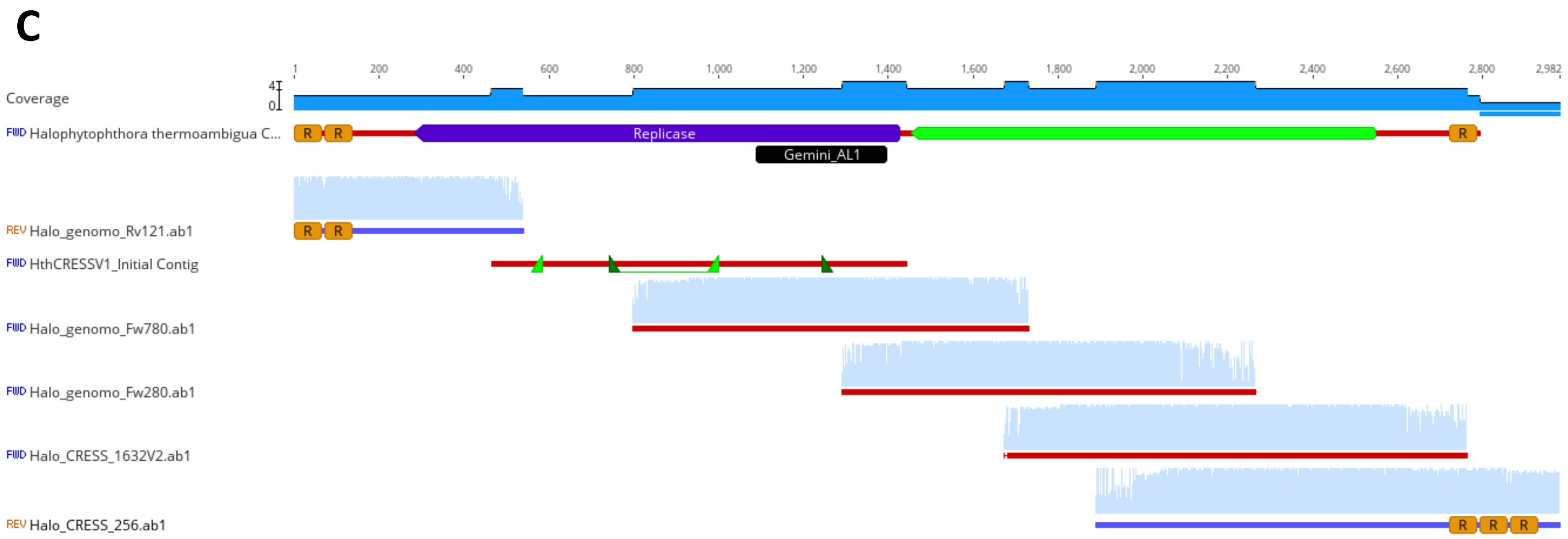

Figure S1. **A,** Gel picture with the amplification of the initial contig using different primer combinations: (1) Halo_DNA_280F X Halo_DNA_536R, (2) Halo_DNA_780F X Halo_DNA_121R, (3) Halo_DNA_280F X Halo_DNA_121R, (4) Halo_DNA_280F X Halo_DNA_121R (M). DNA size Marker
B, Picture of the primer names and sites designed in the original contig. **C,** Coverage Plots illustrating the amplicon-derived sequences in Geneious Prime. **D,** Coverage Plot illustrating the read
sense in Geneious Prime (next slide)

Figure S1. D Coverage Plot illustrating the read reassembling to the final sequence of HathCRESSV1 in Geneious Prime

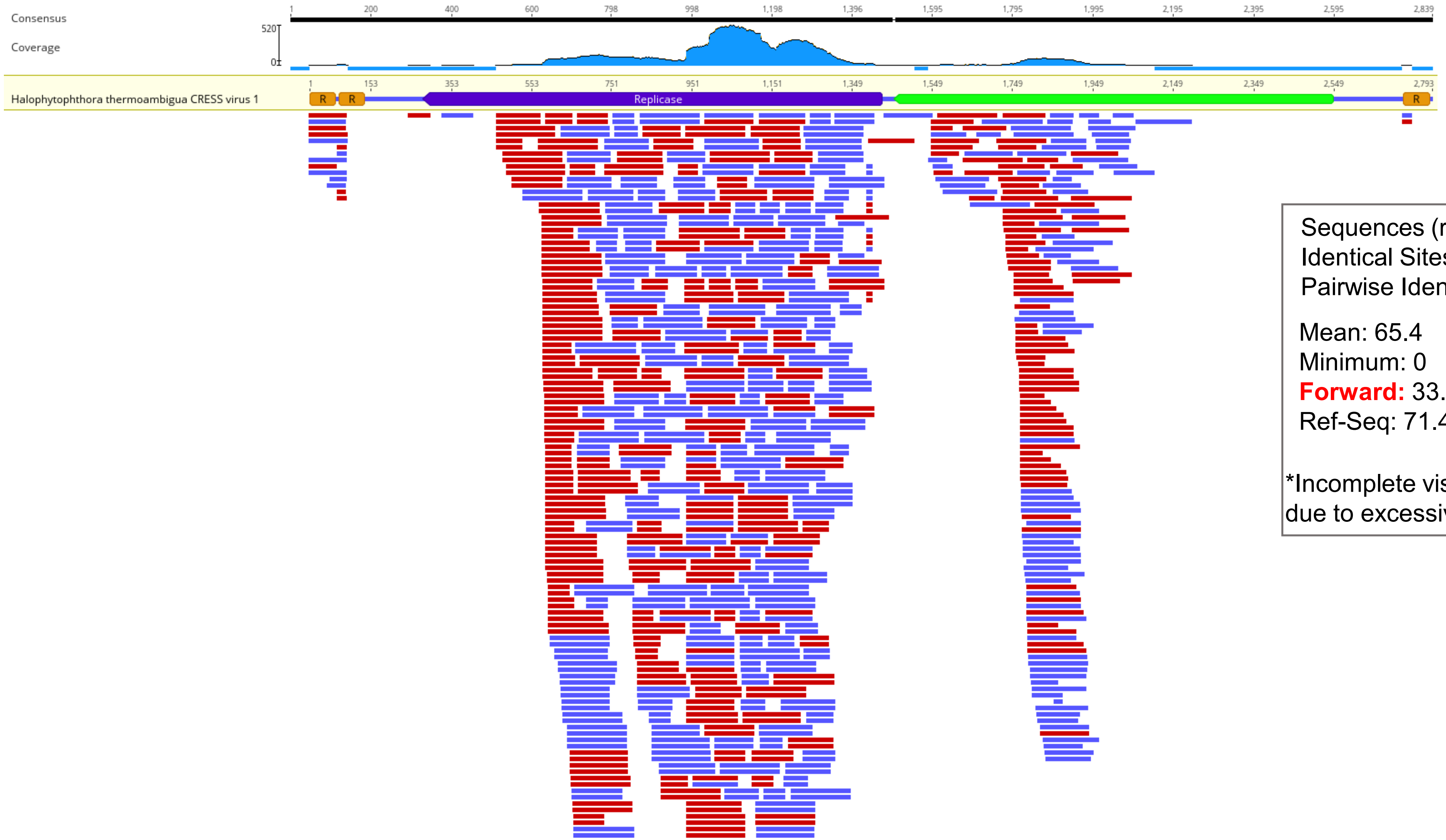

Sequences (reads): 1,637
Identical Sites: 1,847 (92.4%)
Pairwise Identity: 98.9%

Mean: 65.4 Std Dev: 116.2
Minimum: 0 Maximum: 520
Forward: 33.3 **Reverse:** 32.1
Ref-Seq: 71.4% (1,995 of 2,793)

*Incomplete visualisation of the alignment due to excessive depth

Supplementary Figure S2. Predicted secondary structures of putative stem–loop motifs in the HthCRESSV1 genome.

A

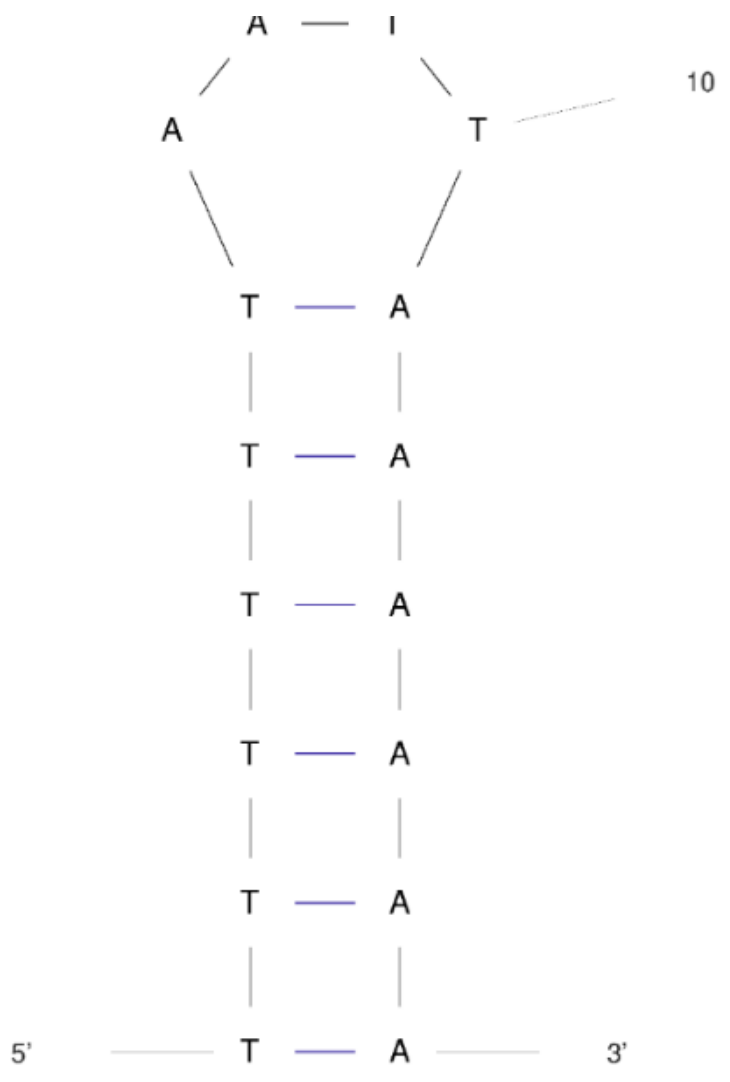

Genomic positions 1,433 – 1,448 nt. The free energy of the thermodynamic ensemble is -2.28 kcal/mol.
The frequency of the MFE structure in the ensemble is 33.15 %.
The ensemble diversity is 6.59

B

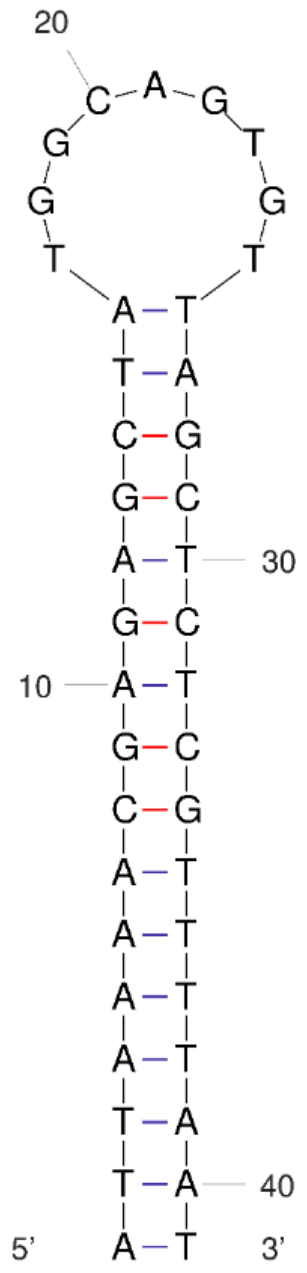

Genomic positions 214 – 254 nt. The free energy of the thermodynamic ensemble is -15.07 kcal/mol. The frequency of the MFE structure in the ensemble is 64.11 %.
The ensemble diversity is 0.86 .

C

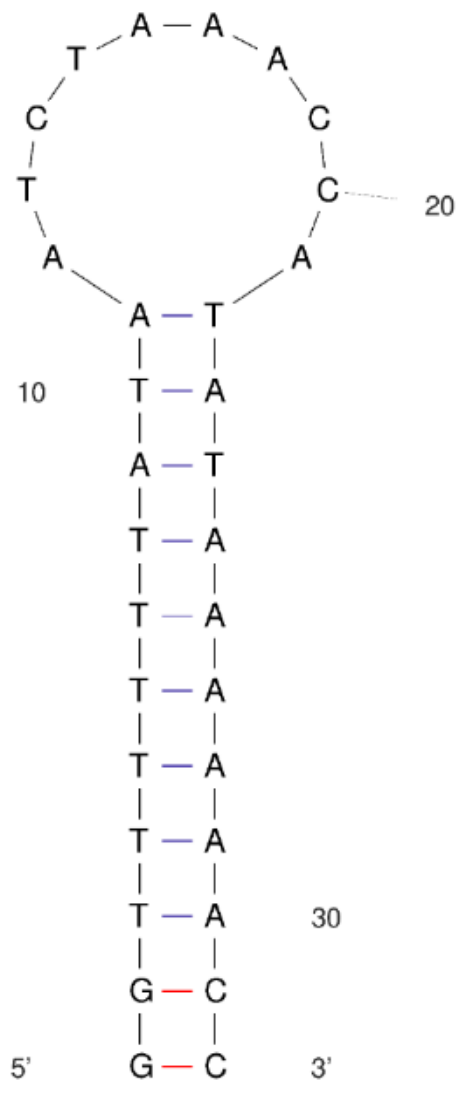

Genomic positions 57 – 88 nt and 2,778 - 2,809 nt (16 – 2,778 nt).

The free energy of the thermodynamic ensemble is -6.73 kcal/mol.
The frequency of the MFE structure in the ensemble is 49.39 %.
The ensemble diversity is 1.91 .

Secondary structures were predicted for selected genomic regions using minimum free energy folding models under DNA parameters. The analyzed regions include the SIR (**A**) and two internal genomic fragments **(B–C)**. All structures display stable stem–loop conformations, suggesting potential roles in genome replication or regulation. Nucleotide positions are indicated relative to each analyzed fragment.

Supplementary Figure S3. Amino acid alignment showing conserved motifs in the hypothetical protein of HthCRESSV1

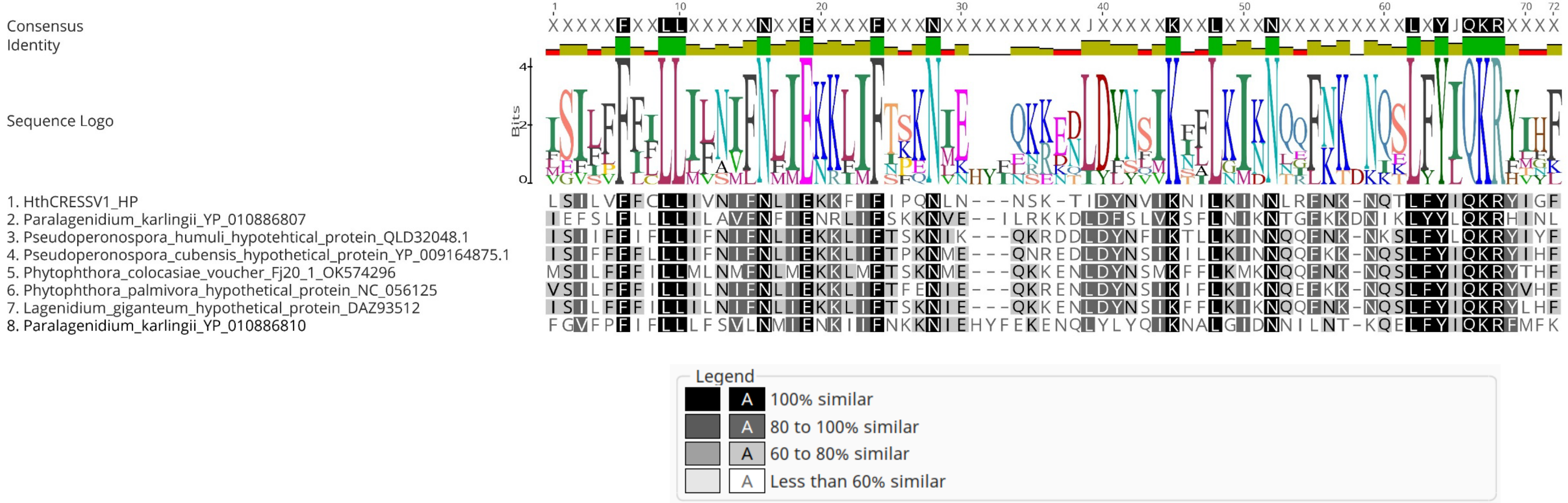

Supplementary Figure 4S. Structural modelling of the oligomers of the HP ectodomain

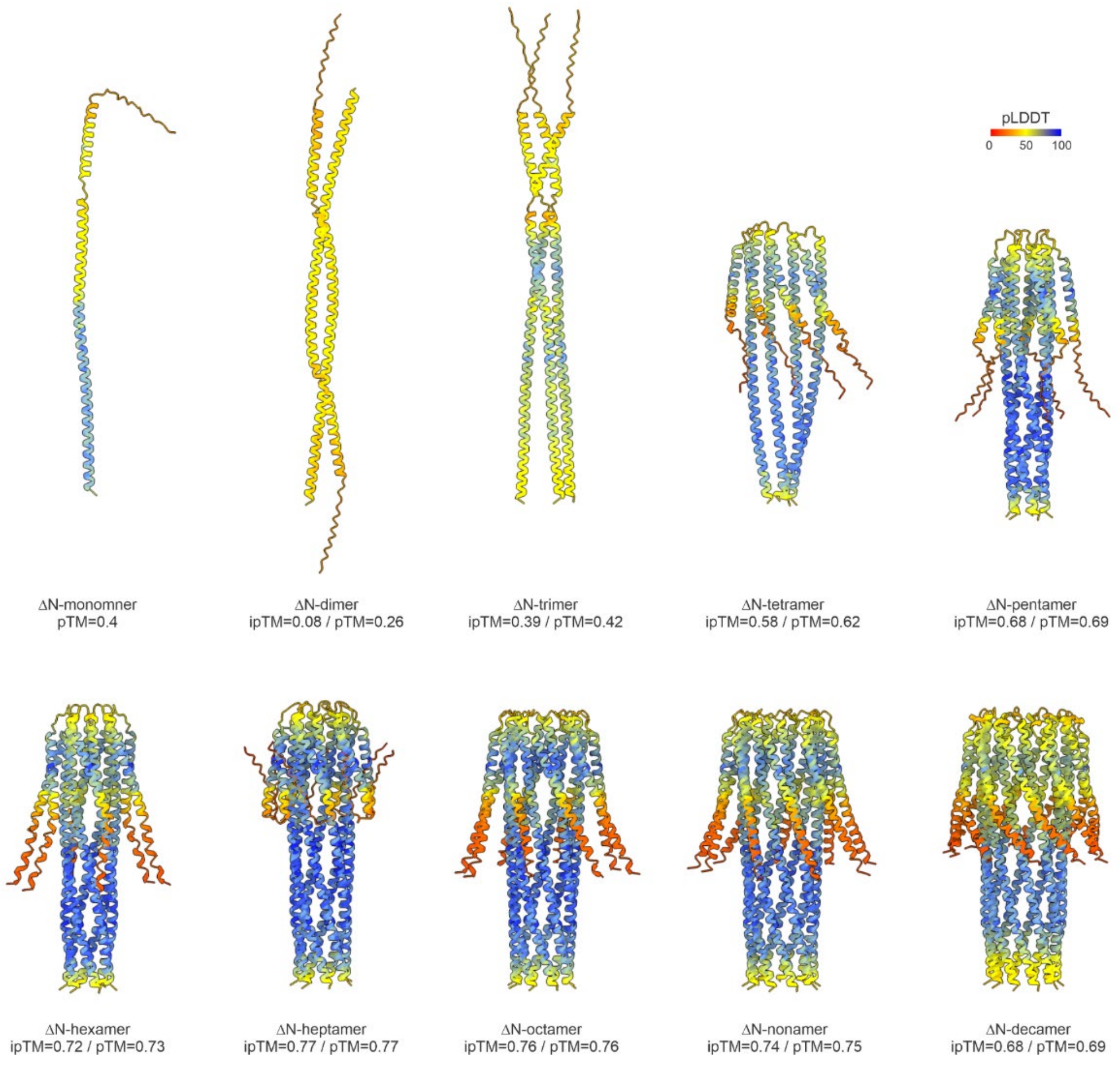

The figure shows multimeric states of the HP ectodomain from monomer to decamer colored based on the predicted local distance difference test (pLDDT) score, a per-residue measure of local confidence, from red to blue (the color scale is provided in the top right corner of the figure). The predicted template modelling (pTM) and the interface predicted template modelling (ipTM) scores are also indicated under each model.

Supplementary Figure S5. Attempts to eliminate HthCRESSV1 from strain BD651.

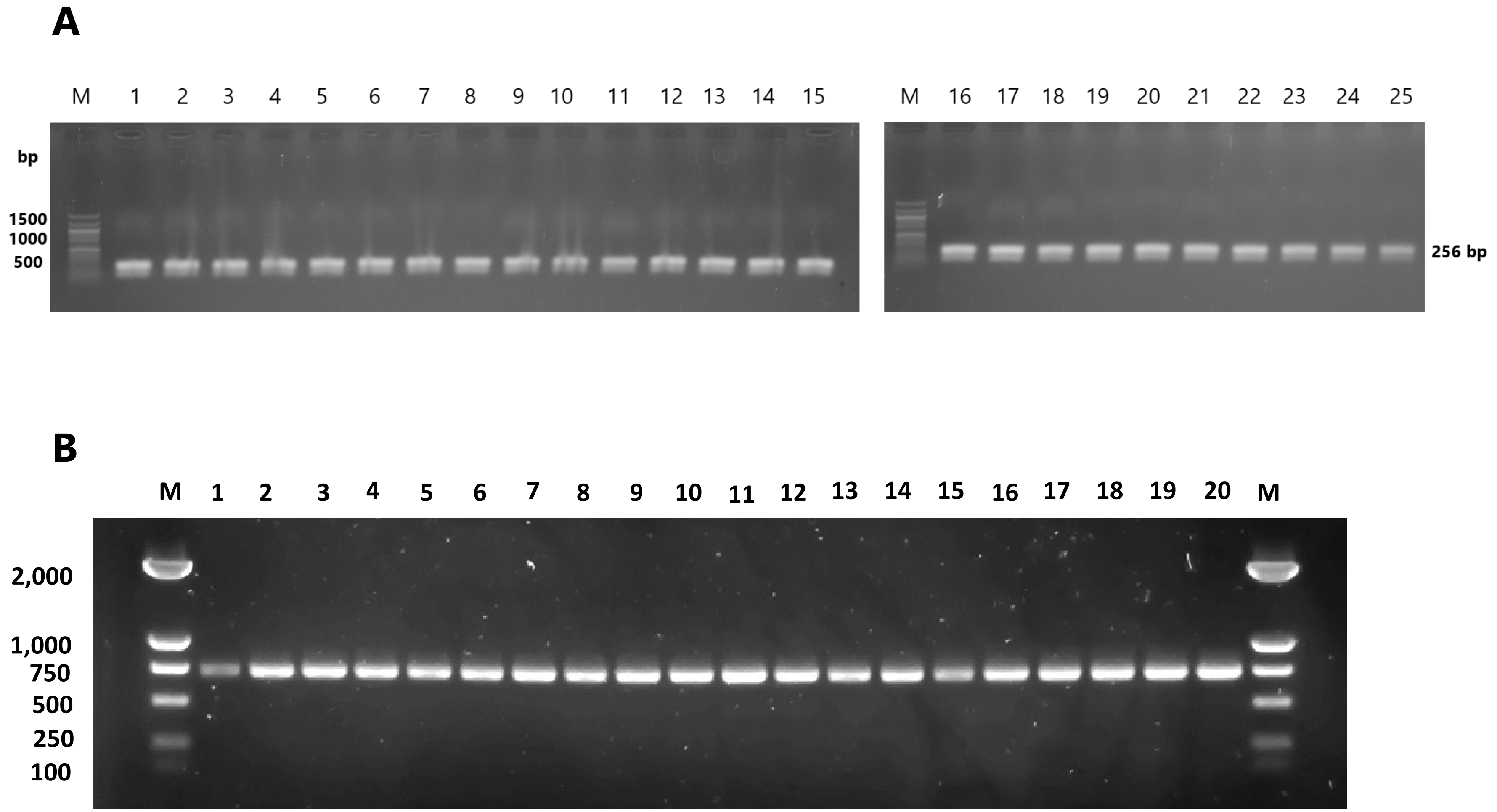

(A) PCR detection of HthCRESSV1 in 25 strains obtained by single zoospore isolation. M, DNA size marker. **(B)** PCR detection of HthCRESSV1 in protoplast-regenerated strains exposed to ribavirin (300 µg ml^{-1} final concentration) and cycloheximide (5 µg ml^{-1} final concentration) in regeneration medium. Regenerated mycelia were subsequently transferred to s. V8A medium without ribavirin and cycloheximide. M, DNA size marker.
